# Predicting women’s intention to use contraceptives in East Africa: a machine learning analysis of predictors

**DOI:** 10.1186/s12905-026-04705-7

**Published:** 2026-07-20

**Authors:** Andualem Addisu Birlie, Tamir Wondim Desta, Biruktawit Lelisa Eticha, Adisu Meles Kabtyimer, Altaseb Beyene Kassaw, Halid Worku Jemil, Tesfaye Deribe Bedada

**Affiliations:** 1 Department of Health Informatics, College of Medicine and Health Science, Samara University, Samara, Ethiopia; 2 Department of Health Informatics, Arba Minch Health Science College, Arba Minch, Ethiopia; 3 Department of Health Informatics, School of Public Health, College of Medicine and Health Science, Wachemo University, Hosanna, Ethiopia; 4 Department of Epidemiology and Biostatistics, School of Public Health, College of Medicine and Health Sciences, Wollo University, Dessie, Ethiopia; 5 Department of Biomedical Science, College of Medicine and Health Science, Wollo University, Dessie, Ethiopia; 6 Department of Health Informatics, School of Public Health, College of Medicine and Health Science, Wollo University, Dessie, Ethiopia; 7 Department of Health Informatics, School of Public Health, Institute of Health, Bule Hora University, Bule Hora, Ethiopia

**Keywords:** Women, Machine learning, Intention to use, Contraceptive, East Africa, DHS

## Abstract

**Introduction:**

Intention to use contraceptives reflects an individual’s or couple’s plan to adopt contraceptive methods, supporting women’s reproductive autonomy. It is associated with reduced unintended pregnancies, unsafe abortions, and high fertility rates, thereby improving maternal and infant health outcomes. Machine learning approaches can strengthen prediction accuracy and support evidence-based reproductive health planning. This study aimed to predict women’s intention to use contraceptives in East Africa using machine-learning methods.

**Methods:**

We analyzed Demographic and Health Survey (DHS) data from 11 East African countries (2015–2024) in a community-based cross-sectional design. Country-specific sampling weights, stratification, and clustering were applied to account for the complex survey design. Missing data were imputed using the KNNImputer, and predictors were harmonized across surveys. Data preprocessing included cleaning, transformation, integration, and one-hot encoding, with an 80/20 train-test split. Seven machine learning algorithms were evaluated: adaptive boosting, CatBoost, random forest, light gradient boosting, extreme gradient boosting, logistic regression, and decision tree. Hyperparameters for CatBoost were tuned using Bayesian optimization under stratified 10-fold cross-validation. Model transportability was assessed using leave-one-country-out cross-validation.

**Results:**

Among 123,290 reproductive-age women in East Africa, 51.23% reported intention to use contraceptives. Boosted tree algorithms performed best, particularly CatBoost achieving the highest discrimination with an AUC of 80.09% and an accuracy of 73.48%. Leave-one-country-out cross-validation confirmed moderate transportability with an AUC 74%, while calibration analysis showed reliable probability estimates (Brier score 0.179). SHAP feature importance identified employment, education, age, hearing about family planning, pregnancy, breastfeeding, barriers to healthcare access, and fertility preference as the most influential predictors of contraceptive intention.

**Conclusion:**

This study provides a transparent, reproducible framework for pooled DHS prediction modeling, offering actionable insights for policymakers and health planners while serving as a methodological foundation for future applied work.

**Supplementary Information:**

The online version contains supplementary material available at https://doi.org/10.1186/s12905-026-04705-7.

## Introduction

Contraception refers to the intentional use of methods, medications, surgical procedures, or devices to prevent pregnancy [1]. The intention to use a contraceptive is defined as an individual’s or couple’s plan to adopt contraceptive methods, which have important health benefits, including reduced fertility rates and unmet contraceptive needs, ultimately improving maternal and infant health outcomes [2, 3]. Access to comprehensive contraceptive options not only reduces unwanted pregnancies and unsafe abortions but also saves lives: in 2017 alone, 308 million unintended pregnancies were prevented, maternal deaths fell from 308,000 to 84,000, and newborn deaths declined from 2.7 million to 538,000 [4–7].

Despite these benefits, sub-Saharan Africa (SSA) countries report the lowest contraceptive prevalence (27.8%), compared to the global average of 49.0% [8]. National demographic and health surveys (DHS) report wide variation in contraceptive intention across countries. For example, prevalence was 31.70% in Ghana [9], 44.70% in Mozambique [10], 44.11% in Ethiopia [11], 35.0% in Benin [4], 39.42% in Liberia [12], and 29% in Nigeria [5]. Within sub-Saharan Africa overall, DHS data indicate an average prevalence of 37.7%, with country-specific estimates such as 42.3% in Niger, 37.9% in Mali, 37.6% in the Democratic Republic of Congo, 31.3% in Angola, 20.6% in Gambia, and 20.3% in Chad [13]. These national-level comparisons highlight regional heterogeneity in contraceptive intention.

Prior studies have largely described prevalence and correlates of contraceptive intention, identifying demographic, socioeconomic, and behavioral predictors such as age, education, marital status, place of residence, employment status, sex of household head, wealth index, number of children, media exposure, fertility preference, and healthcare access [4, 11, 12, 14–16]. However, most analyses remain descriptive or rely on traditional regression models. Regression remains valid, but it is limited in capturing nonlinear relationships and complex interactions in large, multidimensional datasets, such as DHS [17–19]. This creates a clear scientific gap: few studies have advanced to predictive modeling that can identify which women are most likely to intend contraceptive use. Without such models, policymakers lack actionable tools to target interventions and allocate resources efficiently. Machine learning methods, by contrast, are designed for prediction-oriented objectives, can flexibly accommodate nonlinearities and high-dimensional feature spaces, and provide variable importance measures that highlight the most influential predictors [20, 21].

Therefore, the objective of this study was to develop a predictive model of women’s intention to use contraceptives in East Africa (2015–2024) using machine learning approaches and to identify the most influential predictors. This study provides a transparent, reproducible framework for pooled DHS prediction modeling, offering actionable insights for policymakers and health planners while serving as a methodological foundation for future applied work.

## Methods

### Study design, setting, and period

In this study, the data were collected based on a cross-sectional study design in eleven East African countries using the DHS dataset collected from 2015 to 2024. East Africa is a sub-region of Africa that comprises twenty internationally recognized countries. Among those countries, this study used standardized DHS data from eleven East African countries, including Burundi, Ethiopia, Kenya, Madagascar, Malawi, Mozambique, Rwanda, Tanzania, Uganda, Zambia, and Zimbabwe, as shown in Table 1. Based on information from the East African DHS, this study employed a predictive modeling approach to predict the intention of reproductive-age women to use contraceptives.

**Table 1:** The number of study participants in each East African country

Country	Year	Number of non-contraceptive user reproductive age women			Percentage
		Not intended	Intended	Total	
Burundi	2016/17	5,802	8,378	14,180	11.50
Ethiopia	2016	5,238	6,470	11,708	9.50
Kenya	2022	12,724	4,445	17,169	13.92
Madagascar	2021	5,838	5,471	11,309	9.18
Malawi	2015/16	3,992	9,284	13,276	10.77
Mozambique	2022/23	7,072	2,707	9,779	7.93
Rwanda	2019/20	2,711	6,275	8,986	7.29
Tanzania	2022	6,944	3,592	10,536	8.55
Uganda	2016	5,085	7,819	12,904	10.47
Zambia	2024	3,292	5,030	8,322	6.75
Zimbabwe	2015	1,427	3,694	5,121	4.15
Total		60,125	63,165	123,290	100.00

### Data sources, sample size determination, and sampling technique

This study is a secondary analysis of DHS data. We used the Women’s Record (IR file) and other DHS program datasets that are publicly accessible online. Countries were included in the pooled analysis if they met three eligibility criteria. First, a DHS survey had to be conducted between 2015 and 2024 to ensure recency and comparability across settings. Second, the dataset needed to contain the relevant outcome variable, namely contraceptive intention among women aged 15–49. Third, the dataset had to provide complete and consistently coded predictor variables as defined by DHS guidelines. The analytic sample consisted of non-contraceptive-using women of reproductive age (15–49 years) drawn from DHS surveys conducted between 2015 and 2024 in 11 East African countries. Current contraceptive users were excluded to focus on women’s intention to initiate contraceptive use. Infecund women, defined by DHS as those reporting inability to conceive, were also excluded because contraceptive intention is not relevant for them. Women who reported being “unsure” about future contraceptive use were recoded as having “no intention,” in line with DHS analytic conventions. This approach yielded a final pooled sample of 123,290 women.

DHS surveys employ a two-stage-stratified cluster sampling design. In the first stage, enumeration areas (EAs) were selected using stratified random sampling. In the second stage, households within selected EAs were chosen using systematic random sampling. All eligible women in selected households were interviewed using standardized DHS questionnaires. To account for the complex survey design, we applied country-specific sampling weights, stratification, and clustering at the primary sampling unit (PSU) level. When pooling across countries, weights were rescaled to reflect each country’s population size relative to the pooled dataset. Weighted estimates were used for descriptive statistics and model evaluation to ensure representativeness.

### Variables of the study

The dependent variable was women’s intention to use contraceptives among non-users of reproductive age. This variable was derived from the DHS item: “Do you intend to use a method to delay or avoid pregnancy in the future?” Response options were “Yes,” “No,” and “Unsure/Don’t know.” For analysis, the variable was dichotomized as 1 = Intended and 0 = Not intended. Women who responded “Unsure/Don’t know” were recoded as “Not intended.” Current contraceptive users and infecund women were excluded, consistent with DHS guidelines [4, 11, 12, 14–16].

Independent variables were selected a priori based on DHS program standards and prior literature. In addition, we included some predictors available in DHS, specifically current breastfeeding status, barriers to healthcare access, and contact with a field worker in the past 12 months, to test their association with contraceptive intention. The final predictor set included age, religion, educational status, place of residence, employment status, marital status, sex of household head, wealth index, media exposure, current pregnancy status, current breastfeeding status, heard about family planning, knowledge of any contraceptive method, barriers to healthcare access, fertility preference, ideal number of children, contact with a field worker in the past 12 months, visit to a health facility in the past 12 months, and number of living children [4, 9–12, 14–16].

### Variable harmonization

Variable harmonization was conducted following the DHS recode manuals. All 19 predictors and the outcome variable were defined using standardized DHS variable names, which are consistent across surveys and countries. Composite indicators (e.g., barriers to healthcare access, media exposure, and hearing about family planning) were constructed according to DHS guidelines by grouping relevant items. No variables required ad hoc recoding, and all predictors were harmonized across the 11 DHS surveys. Therefore, all predictors are defined and coded uniformly across DHS surveys.

### Operational definition

#### Media exposure

Defined as regular contact with mass media to access general information. Women were classified as exposed if they reported watching television, listening to the radio, or reading newspapers/magazines at least once per week.

#### Heard about family planning

Defined as having heard family planning messages through television, radio, newspapers, or magazines, specifically to capture targeted health communication. Women were classified as exposed if they answered “yes” to at least one of these items.

#### Barriers to healthcare access

The composite barriers to healthcare access are classified as “yes” if the respondent reports encountering at least one of the following difficulties: getting authorization to seek treatment, obtaining funding for treatment, the distance to the medical facility, or not wanting to travel alone, and “no” if none of these difficulties were mentioned.

### Heterogeneity, subgroup, and sensitivity analyses

We assessed heterogeneity across all 11 countries to evaluate variability in model performance and to identify potential outliers. Subgroup analyses were conducted by stratifying the dataset into two survey periods, 2015–2020 and 2021–2024, to examine temporal consistency. Sensitivity analyses were performed at the country level, with models iteratively evaluated across individual countries to test robustness and transportability. These complementary analyses enhance transparency and provide a more nuanced understanding of model generalizability across diverse national contexts.

### Data management and statistical analysis

#### Data preprocessing

**Fig. 1:**
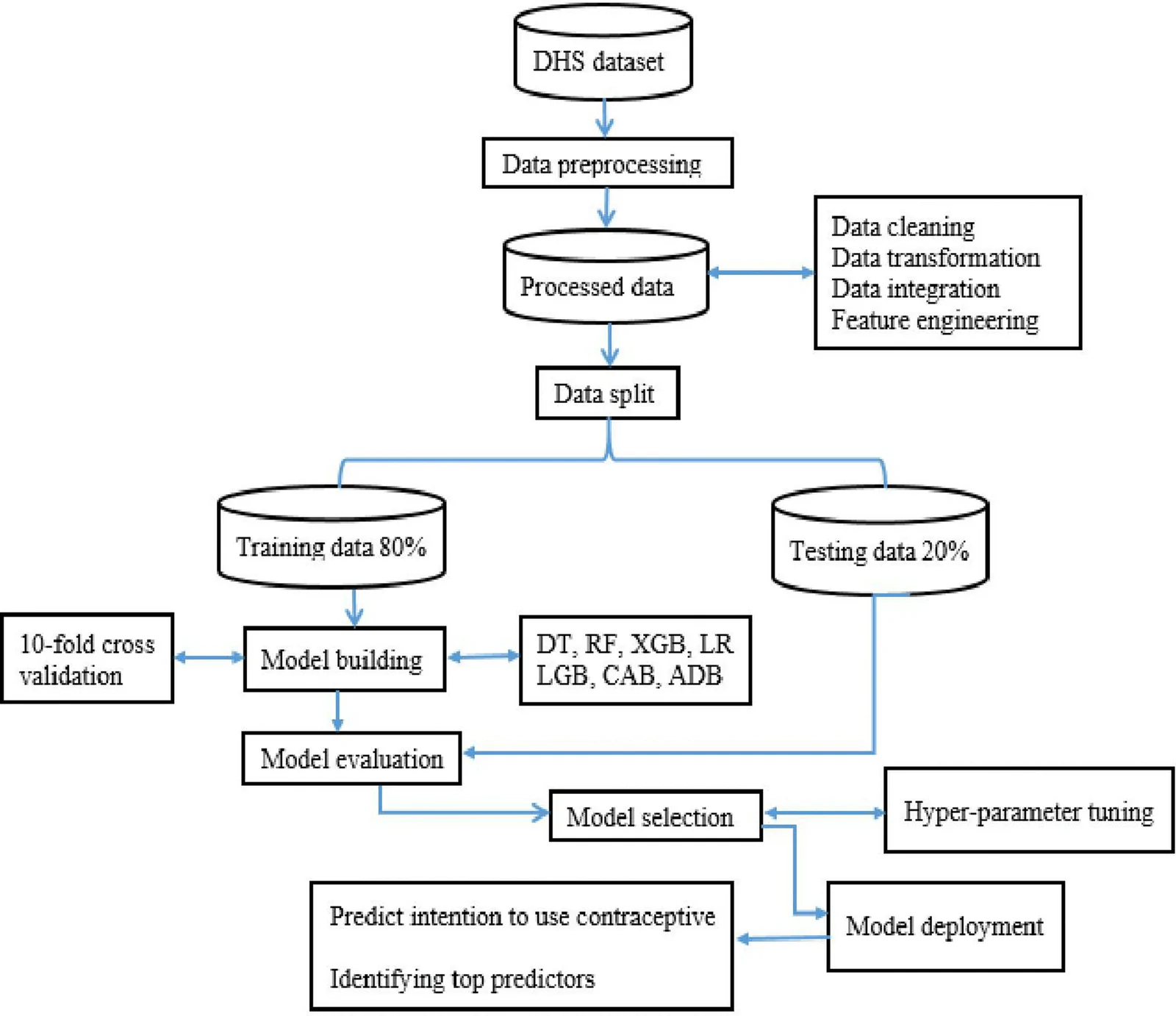
Summary of data preparation and analysis process plan

Initial exploratory checks for outliers, missing values, and consistency were performed using Microsoft Excel 2019 and STATA/MP version 17. Descriptive statistics were summarized in tables and graphs as frequencies and percentages. However, all final data cleaning and preprocessing steps were conducted in a fully scripted, reproducible workflow using Python (version 3.12) in Jupyter Notebook, which was used for machine learning analysis utilizing packages like Pandas (v2.2.2), Seaborn (v0.13.2), Scikit-Learn (v1.4.2), and SHAP (v0.50.0). The machine learning analyses were carried out using the six steps of the machine learning algorithms, which are data collection, preparation, model selection, training, evaluation, and prediction, as shown in Fig. 1. Handling missing values, standardization, categorical encoding, feature selection, and dimensionality reduction were among the preprocessing procedures applied to the data. Before imputation, we examined the extent of missingness across all variables. Most predictors had complete data, but several variables contained missing values: visited by a field worker in the last 12 months (7%); visited a health facility in the last 12 months (7%); fertility preference (7%); ideal number of children (10.5%); and religion (8.6%). All other variables had no missing values. The missing values were imputed using the KNNImputer technique from scikit-learn. This method replaces missing entries by averaging values from the k nearest neighbors in the dataset, thereby preserving multivariate relationships among predictors. One-hot encoding was used to create dummy variables for categorical variables. To prevent information leakage in model evaluation, all reported performance metrics were based on cross-validation procedures in which imputation and encoding were fit only on the training folds and then applied to the validation folds. This design ensured that no information from the test data influenced model training. Before modeling, we assessed collinearity using variance inflation factors (VIFs) and pairwise correlations. No variables exceeded conventional thresholds (VIF > 10) and were found between 1.01 and 8.59.

### Model selection and development

To balance interpretability and predictive power, seven classification algorithms were chosen: Adaptive Boosting (AdaB), Cat Boosting (CatB), Light Gradient Boosting (LGB), Extreme Gradient Boosting (XGB), Random Forest (RF), Decision Tree (DT), and Logistic Regression (LR) [22]. While ensemble approaches (random forest, XGB, LGB, CatB, and AdaB) were given priority due to their capacity to handle high-dimensional data, model nonlinear relationships, and prevent overfitting through bagging and boosting mechanisms [23]. Simpler models (e.g., decision trees and logistic regression) were used to evaluate trade-offs in complexity [24]. We employed an 80:20 train-test split to evaluate the models’ performance, allocating 80% of the dataset for training and the remaining 20% for testing. Because the train-test split function method has the issue of either overfitting or underfitting on the dataset, the standard k-fold cross-validation methodology was employed to ensure the model’s performance [25]. Within the training set, 10-fold stratified cross-validation was applied to estimate model performance and tune hyperparameters. The final model was then evaluated on the independent 20% test set, which was not used during training or cross-validation. This design ensured that cross-validation occurred only within the training set and that the test set provided an unbiased estimate of generalization. In addition to stratified 10-fold cross-validation, we employed a leave-one-country-out cross-validation (LOCO-CV) design to evaluate model transportability across diverse national contexts. In this framework, the dataset was partitioned by country, and in each iteration, the model was trained on all but one country and tested on the held-out country. Performance metrics, including AUC, accuracy, precision, recall, and F1 score, were computed for each held-out country. The overall summary statistics (mean ± SD) across all countries were then reported to provide a concise measure of generalizability. These complementary approaches demonstrate that our models are not only internally consistent but also externally robust, while transparently revealing heterogeneity across countries and survey waves, thus providing a more policy-relevant assessment of regional applicability.

### Performance metrics

We employed an extensive set of performance metrics to assess the models’ efficacy, guaranteeing a fair evaluation of predictive accuracy and dependability. Accuracy, area under the curve (AUC), precision, recall, and F1 score were used to assess how well the models performed. In addition to mitigating overfitting and offering a more trustworthy estimate of the model’s generalization performance, these metrics offered a thorough assessment of the model’s accuracy, capacity to correctly classify instances, and overall predictive power [26]. While precision and recall offered more in-depth information, accuracy served as a broad gauge of the models’ overall performance. The model’s capacity to differentiate between classes was also evaluated using the area under the receiver operating characteristic curve (AUC-ROC) [20, 26]. These metrics provided a solid assessment that showed each model’s advantages and possible drawbacks in terms of predicting the intention to use contraceptives.

### Hyperparameter tuning

In this study, we applied Bayesian optimization using the Optuna framework specifically to the CatBoost model, which was identified as the strongest baseline. The search space included learning rate, maximum depth, border count, bagging temperature, number of trees, and L2 regularization. Optimization was guided by the ROC-AUC metric under stratified 10-fold cross-validation, ensuring robust discrimination performance. While other algorithms were evaluated using default parameters, CatBoost was tuned to provide a fair benchmark of the best achievable performance.

### Prediction

Prediction comprises applying the fully trained model to its intended output and using preselected predictors to predict the result variable. The intention to use contraceptives was determined in this study using the top predictors discovered during the analysis. Therefore, the most accurate classifier will be used to determine whether a woman intends to use contraception to a predetermined degree of accuracy. Threshold-dependent measures were interpreted only after specifying the classification threshold. In our analysis, we used a threshold of 0.5, which is the conventional cutoff in binary classification and ensures interpretability. We acknowledge that alternative thresholds (e.g., based on Youden’s J statistic or prevalence) could be considered, but the choice of 0.5 provides a transparent and reproducible baseline for evaluating confusion matrices and related metrics.

### Model interpretation/explanation using SHAP

The Additive Explanations (SHAP) feature significance approach was used to evaluate the relationship between the predictors and the intention to use contraceptives. This method also helped identify the independent factors that are most significant for predicting intention to use contraceptives. A game theory framework is used by SHAP analysis to provide a local or global interpretation and explanation of any prediction made by a machine-learning model [27]. A waterfall plot, which graphically depicts how the impacts of individual features come together to create the final prediction, was utilized to further elucidate these contributions. An understandable view of how different factors interact to shape the outcome is provided by each section of the plot, which shows the precise contribution of a variable, either positive or negative [28]. When combined, the SHAP beeswarm and bar plots offer a potent method for revealing patterns and relationships in the data, improving transparency and dissecting intricate models [29]. This method improves knowledge of the nature of the data while also helping with model validation. This study adhered to the TRIPOD-AI reporting guideline as shown in S1 Table, which provides harmonized standards for transparent reporting of prediction model studies using regression or machine learning methods [30].

### Ethical consideration

This study used secondary data from the Demographic and Health Surveys (DHS) conducted in East African countries between 2015 and 2024. Access to the DHS datasets was obtained through the DHS Program (www.measuredhs.com) after registration and approval of the research proposal. The DHS Program secures informed consent from all participants and ensures strict confidentiality in accordance with the Declaration of Helsinki and national ethical guidelines. Because the datasets are anonymized and de-identified, no additional ethical approval was required for this secondary analysis. In line with local policy, an institutional review exemption was not sought, as the use of publicly available DHS data does not require further approval. Data were accessed and used solely for this study following DHS approval.

## Results

### Characteristics of the respondent

#### Sociodemographic characteristics

At the outset, 194,022 women of reproductive age were identified. Of these, 68,343 were excluded because they were either current contraceptive users or infecund. This left 125,679 eligible participants before weighting. After applying DHS sampling weights (wt = v005/1,000,000), the final analytic sample comprised 123,290 women. Within this weighted sample, 88,622 (71.88%) resided in rural areas, 48,074 (38.99%) were married, 36,928 (29.95%) were aged 15–19 years, and 80,833 (65.56%) lived in households headed by men, presented in Table 2.

**Table 2:** Sociodemographic characteristics of the respondents

Variables	Categories	Unweighted frequency	Weighted frequency
Residence	Urban	38,641 (30.75)	34,668 (28.12)
	Rural	87,038 (69.25)	88,622 (71.88)
Marital status	Never union	47,802 (38.03)	47,039 (38.15)
	Married	49,819 (39.64)	48,074 (38.99)
	Ever married	28,058 (22.33)	28,176 (22.85)
Women’s age	15–19	37,715 (30.01)	36,928 (29.95)
	20–24	23,431 (18.64)	23,273 (18.88)
	25–29	17,429 (13.87)	17,269 (14.01)
	30–34	14,302 (11.38)	13,849 (11.23)
	35–39	12,358 (9.83)	11,995 (9.73)
	40–44	10,395 (8.27)	10,104 (8.20)
	45–49	10,049 (8.00)	9,868 (8.00)
Sex of household head	Female	44,266 (35.22)	42,457 (34.44)
	Male	81,413 (64.78)	80,833 (65.56)
Religion	Catholic	33,248 (26.45)	34,063 (27.63)
	Protestant	43,406 (34.54)	44,005 (35.69)
	Muslim	14,023 (11.16)	14,149 (11.48)
	Others	35,002 (27.85)	31,072 (25.20)

### Socioeconomic and healthcare access characteristics

From the total study participants, 54,296 (44.04%) had attained primary education, 64,902 (52.64%) were employed, 54,128 (43.90%) belonged to the rich wealth index category, and 110,615 (89.72%) reported media exposure, illustrated in Table 3.

**Table 3:** Socioeconomic characteristics of the respondents

Variables	Categories	Unweighted frequency	Weighted frequency
Women’s education level	No education	25,109 (19.98)	23,193 (18.81)
	Primary	53,961 (42.94)	54,296 (44.04)
	Secondary	39,927 (31.77)	38,715 (31.40)
	Higher	6,682 (5.32)	7,085 (5.75)
Women’s employment status	Unemployed	59,888 (47.65)	58,388 (47.36)
	Employed	65,791 (52.35)	64,902 (52.64)
Wealth index	Poor	48,575 (38.65)	46,235 (37.50)
	Middle	22,486 (17.89)	22,926 (18.60)
	Rich	54,618 (43.46)	54,128 (43.90)
Media exposure	No	12,894 (10.26)	12,674 (10.28)
	Yes	112,785 (89.74)	110,615 (89.72)
Visited the health facility	No	109,250 (86.93)	107,965 (87.57)
	Yes	16,429 (13.07)	15,324 (12.43)
Visited by health workers	No	109,250 (86.93)	107,965 (87.57)
	Yes	16,429 (13.07)	15,324 (12.43)
Barriers to healthcare access	No	56,028 (44.58)	53,396 (43.31)
	Yes	69,651 (55.42)	69,893 (56.69)

### Healthcare access and utilization characteristics

Among the study participants, 73,819 (59.87%) women reported having between one and five children, while 109,416 (88.75%) were not currently pregnant, summarized in Table 4.

**Table 4:** >Healthcare access and utilization characteristics of the respondents

Variables	Categories	Unweighted frequency	Weighted frequency
Numbers of children’s	0	49,779 (39.61)	49,234 (39.93)
	1–5	75,624 (60.17)	73,819 (59.87)
	> 5	276 (0.22)	236 (0.19)
Currently pregnant	No	111,840 (88.99)	109,416 (88.75)
	Yes	13,83 (11.01)	13,874 (11.25)
Heard about family planning	No	73,530 (58.51)	71,916 (58.33)
	Yes	52,149 (41.49)	51,374 (41.67)
Ideal number of children	0	2,791 (2.22)	2,948 (2.39)
	1	1,341 (1.07)	1,384 (1.12)
	2	14,513 (11.55)	14,852 (12.05)
	3	24,887 (19.80)	25,671 (20.82)
	4	36,968 (29.41)	37,561 (30.47)
	5	16,462 (13.10)	15,776 (12.80)
	6+	28,717 (22.85)	25,095 (20.35)
Knowledge of any method	No	4,818 (3.83)	4,128 (3.35)
	Traditional	161 (0.13)	161 (0.13)
	Modern	120,700 (96.04)	119,000 (96.52)
Fertility preference	No	44,760 (35.61)	45,332 (36.77)
	Yes	80,919 (64.39)	77,957 (63.23)
Currently breastfeeding	No	99,772 (79.39)	98,184 (79.64)
	Yes	25,907 (20.61)	25,106 (20.36)

### Pooled prevalence of intention to use contraceptives

**Fig. 2:**
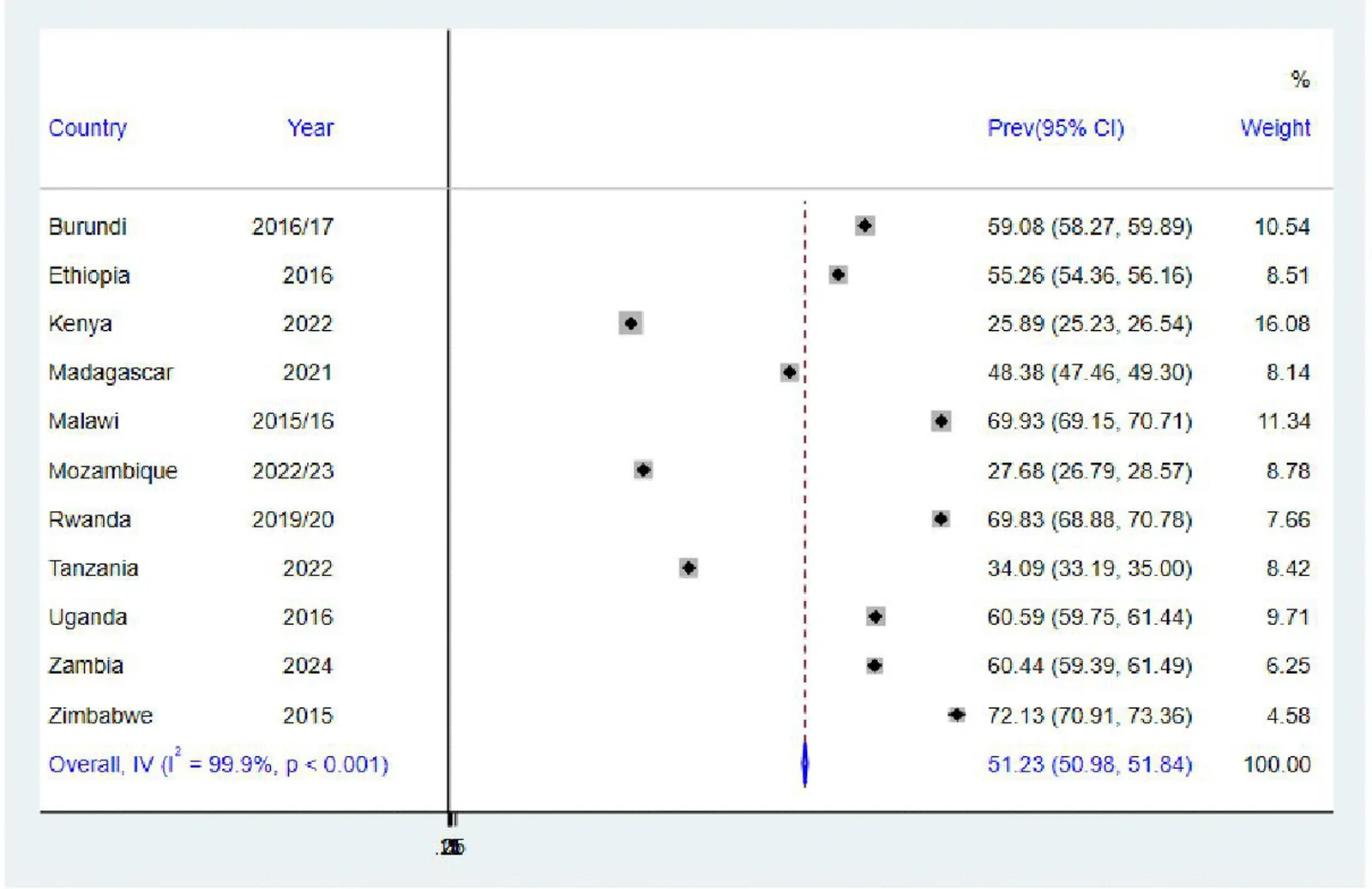
The pooled prevalence of intention to use contraceptives

The pooled prevalence of intention to use contraceptives among reproductive-age women in East Africa was 51.23% (95% CI: 50.98, 51.84), with the lowest proportion in Kenya (25.89%) and the highest proportion in Zimbabwe (72.13%), as shown in Fig. 2. We retained the pooled modeling strategy because pooling ensures adequate sample size and model stability for machine learning algorithms, and our primary aim is to identify individual-level predictors of contraceptive intention across diverse East African populations.

### Heterogeneity of the study

Significant heterogeneity is observed from the random-effects model pooled estimate (I² = 99.9%). To address this heterogeneity, sensitivity and subgroup analyses were performed.

### Sensitivity analysis

**Fig. 3:**
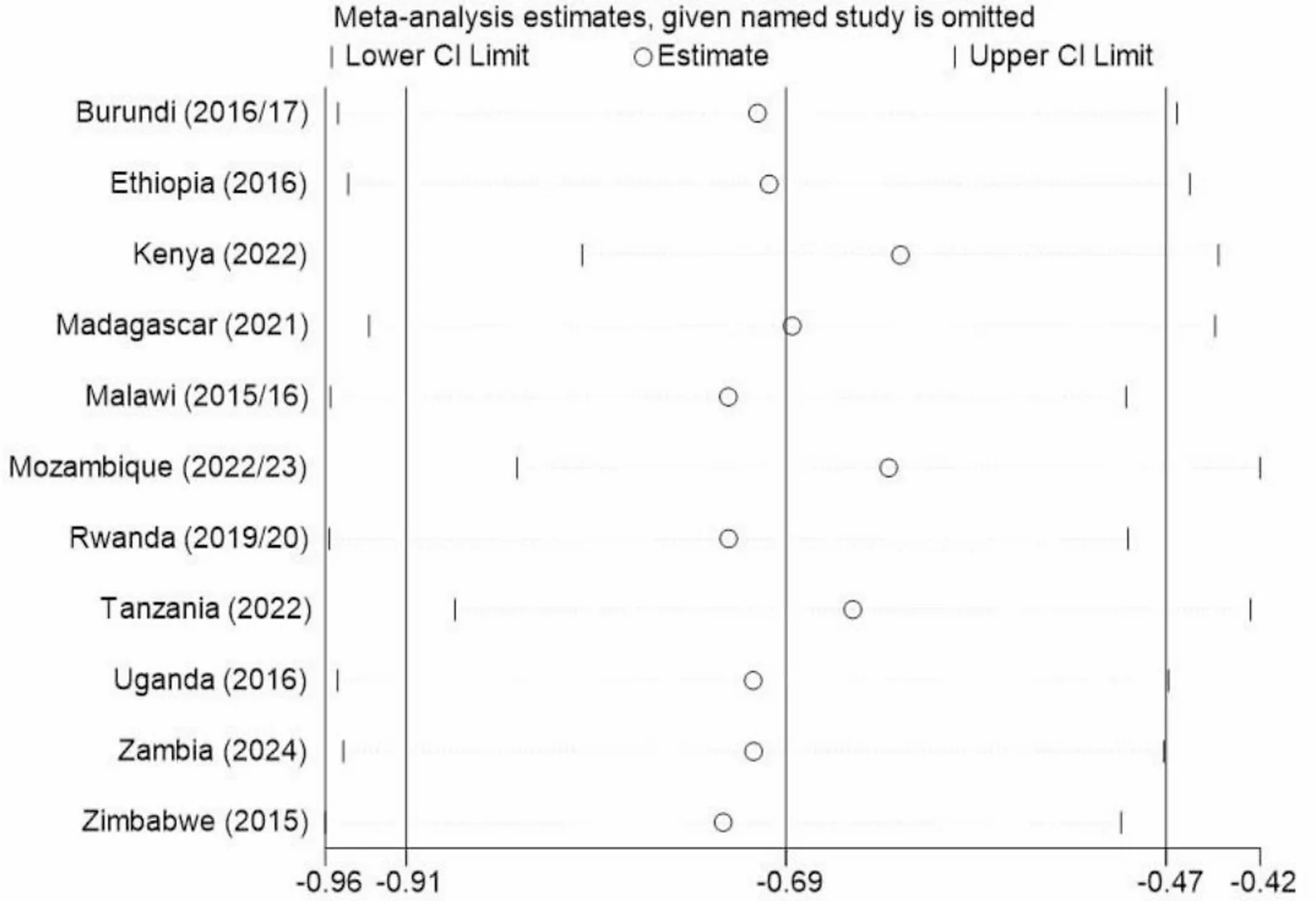
Sensitivity analysis of intention to use contraceptives across East African countries

Sensitivity analyses were conducted to assess the influence of individual countries on the pooled prevalence estimate. When each country was sequentially omitted, the resulting pooled prevalence consistently remained within the 95% confidence interval of the overall estimate. This indicates that no single country disproportionately affected the pooled effect size. Accordingly, the random-effects model confirmed that the overall pooled estimate of contraceptive intention was robust, with no country exerting excessive influence, as illustrated in Fig. 3.

### Subgroup analysis

We performed subgroup analyses by stratifying the DHS survey years into two periods (2015–2020 and 2021–2024) to assess whether temporal variation contributed to the observed.

### Intention to use contraceptives by the DHS survey year

**Fig. 4:**
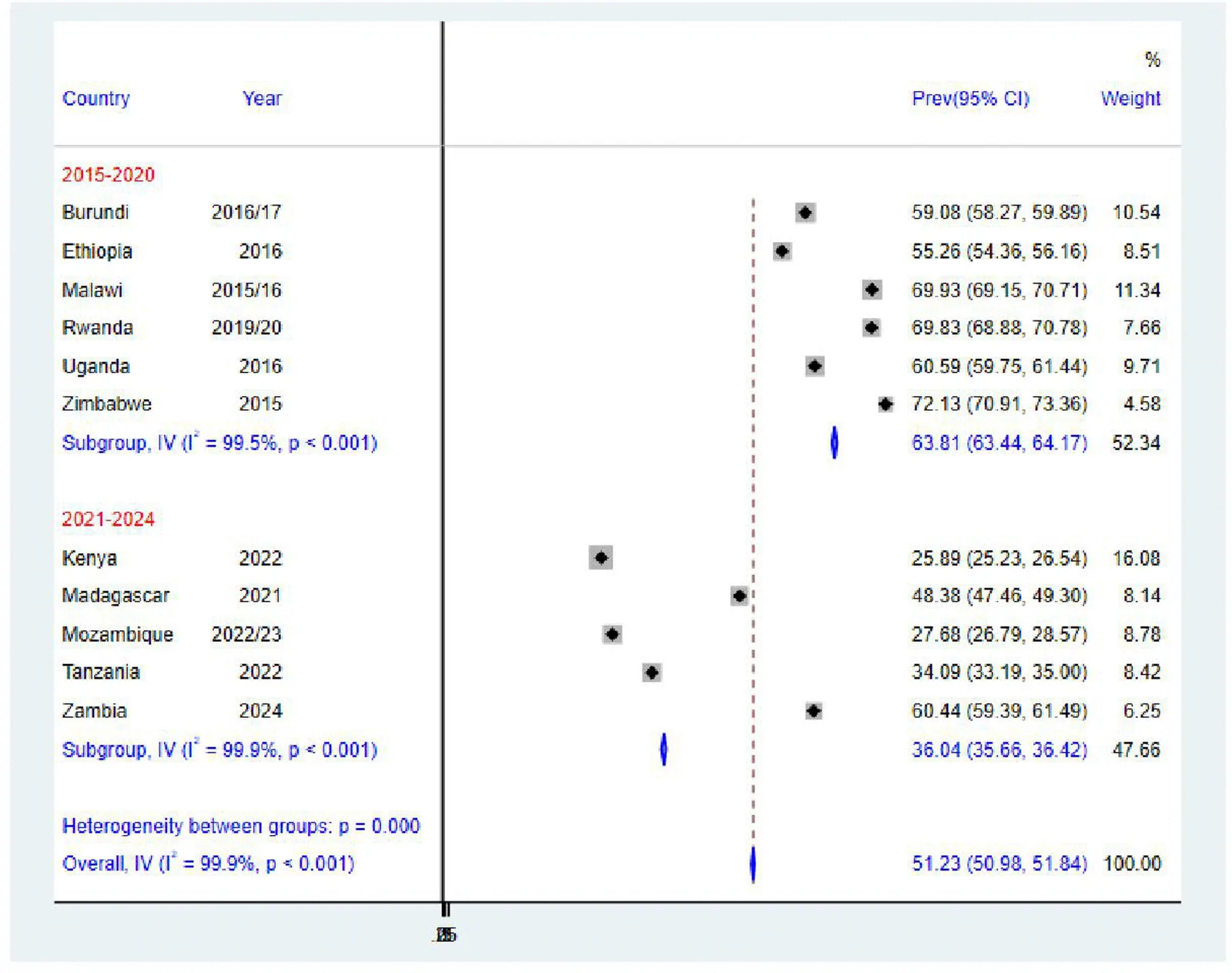
Subgroup analysis of intention to use contraceptives by DHS survey year

According to the results of the subgroup analysis by DHS survey year, the pooled prevalence of intention to use contraceptives was highest (63.81%, 95% CI: 63.44, 64.17) in 2015–2020 and lowest (36.04%, 95% CI: 35.66, 36.42) in 2021–2024. Significant heterogeneity was observed in both periods (2015–2020: I² = 99.5%, *p* < 0.001; 2021–2024: I² = 99.9%, *p* < 0.001) as illustrated in Fig. 4.

### Machine learning result

#### Balancing

**Fig. 5:**
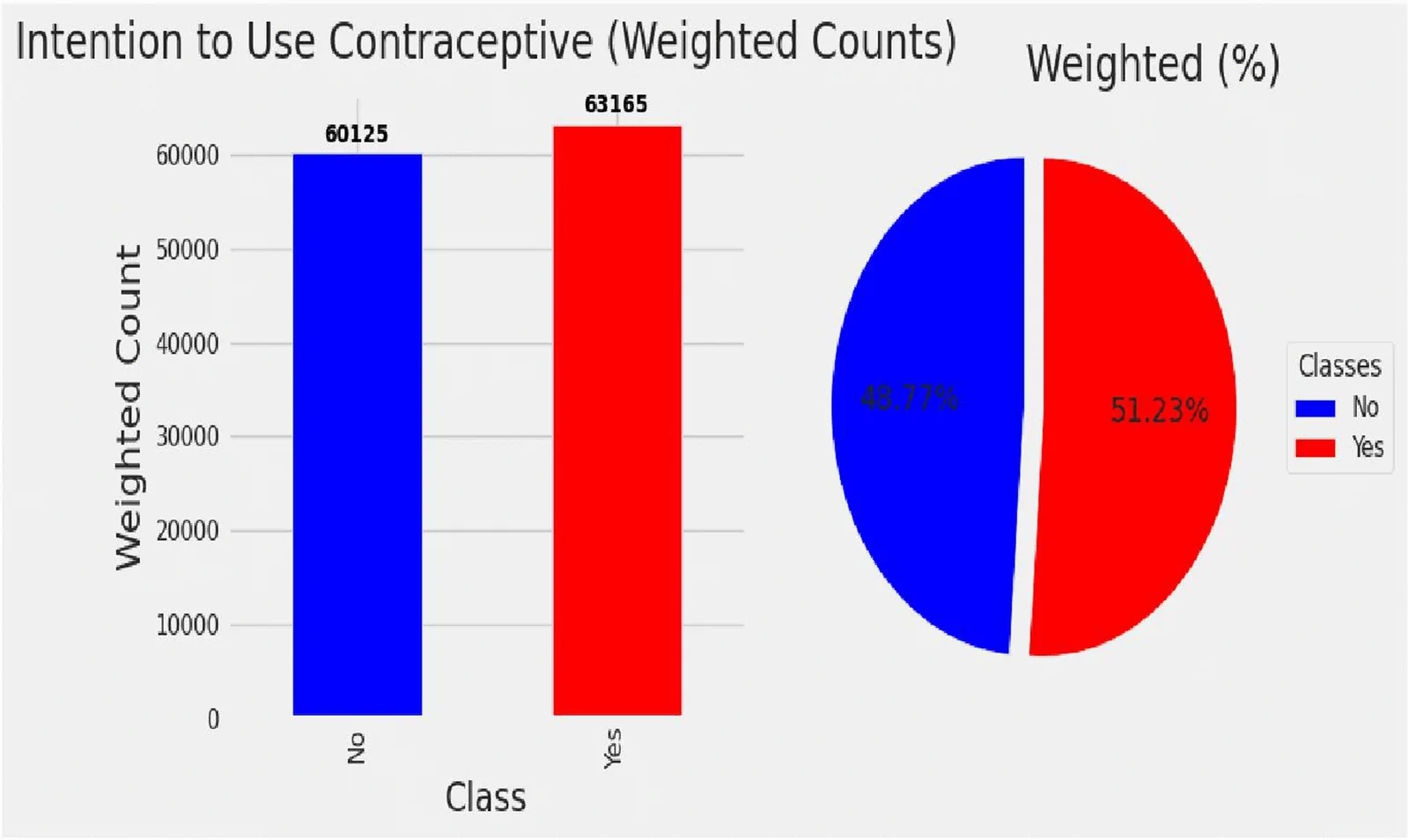
Comparison between intended and not intended to use contraceptive users

We did not use any kinds of balancing techniques because, by default, this dataset was balanced with 63,165 (51.23%) women who intended to use contraception, whereas 60,125 (48.77%) women did not intend to use the contraceptive method presented, as shown in Fig. 5.

#### Model performance

In this study, boosted tree algorithms consistently delivered the strongest predictive performance for modeling contraceptive intention among reproductive-age women. CatBoost achieved the marginally highest values on both the unweighted and weighted samples. The performance of CatBoost on the weighted samples were 80.09% AUC, 73.48% accuracy, 72.22% precision, 79.02% recall, and 75.47% F1-score, as shown in Table 5. To contextualize performance, we compared machine-learning models against logistic regression and the weighted majority class baseline. The weighted majority class baseline achieved 51.6% accuracy. Logistic regression achieved 70.51% accuracy, while CatBoost achieved 73.48%. These comparisons confirm that boosted tree models as a group provided meaningful improvements over traditional baselines, supporting their utility for predictive modeling in contraceptive intention, with CatBoost being only marginally highest in this dataset.

**Table 5:** Comparison of algorithms with different performance metrics

Machine learning model	Performance (%)									
	AUC		Accuracy		Precision		Recall		F1 score	
	Unwt	Wt	Unwt	Wt	Unwt	Wt	Unwt	Wt	Unwt	Wt
Logistic Regression	77.68	77.25	70.78	70.51	68.59	69.09	76.23	77.59	72.21	73.09
Decision Tree	66.51	64.24	65.62	64.22	66.11	65.57	63.49	64.63	64.77	65.10
Random Forest	76.46	77.51	70.40	70.88	69.51	71.02	72.23	73.61	70.84	72.30
Light Boosting	80.02	79.94	73.21	73.22	71.69	72.14	76.33	78.38	73.94	75.13
Cat Boosting	80.08	80.09*	73.09	73.48*	71.34	72.22*	76.82	79.02*	73.98	75.47*
Adaptive Boosting	76.64	75.83	69.83	69.95	68.66	68.89	72.52	76.22	70.54	72.37
Extreme Boosting	79.83	79.89	73.09	73.29	71.38	72.15	76.73	78.61	73.96	75.24

*Unwt* Unweighted sample and *Wt* Weighted sample

*Best performance of the machine-learning algorithm

### ROC curve for the tested models

**Fig. 6:**
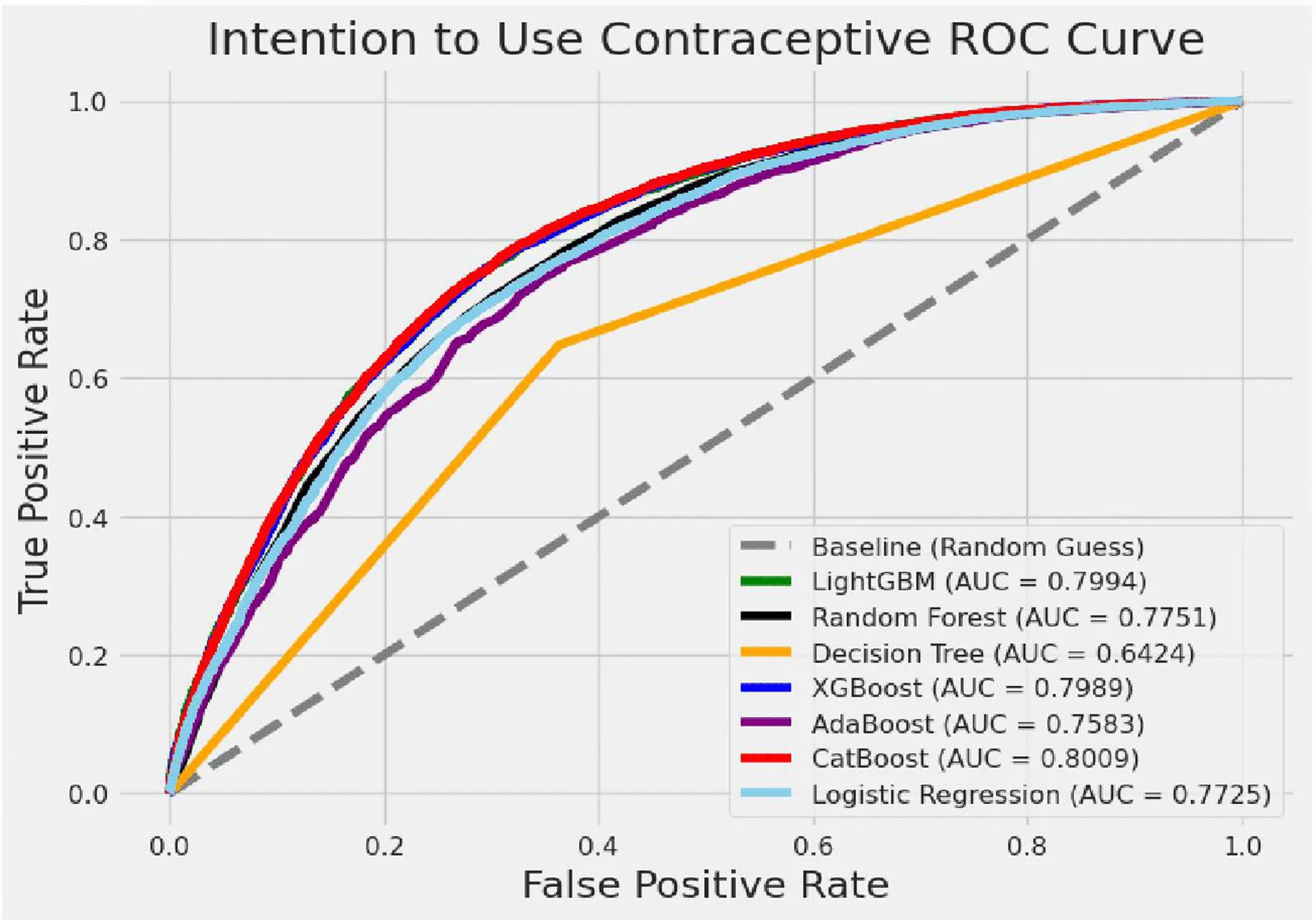
ROC curve for intention to use a contraceptive

The boosted tree models, particularly the CatBoost algorithm, demonstrated the strongest performance among the seven machine learning classifiers evaluated. It achieved an AUC of 0.8009 on the ROC curve, confirming its superior discriminative ability compared to alternative models. This result highlights the robustness of boosted tree methods in handling complex survey data and supports the selection of CatBoost as the optimal predictive model in this study, as illustrated in Fig. 6.

### Hyperparameter tuning

**Fig. 7:**
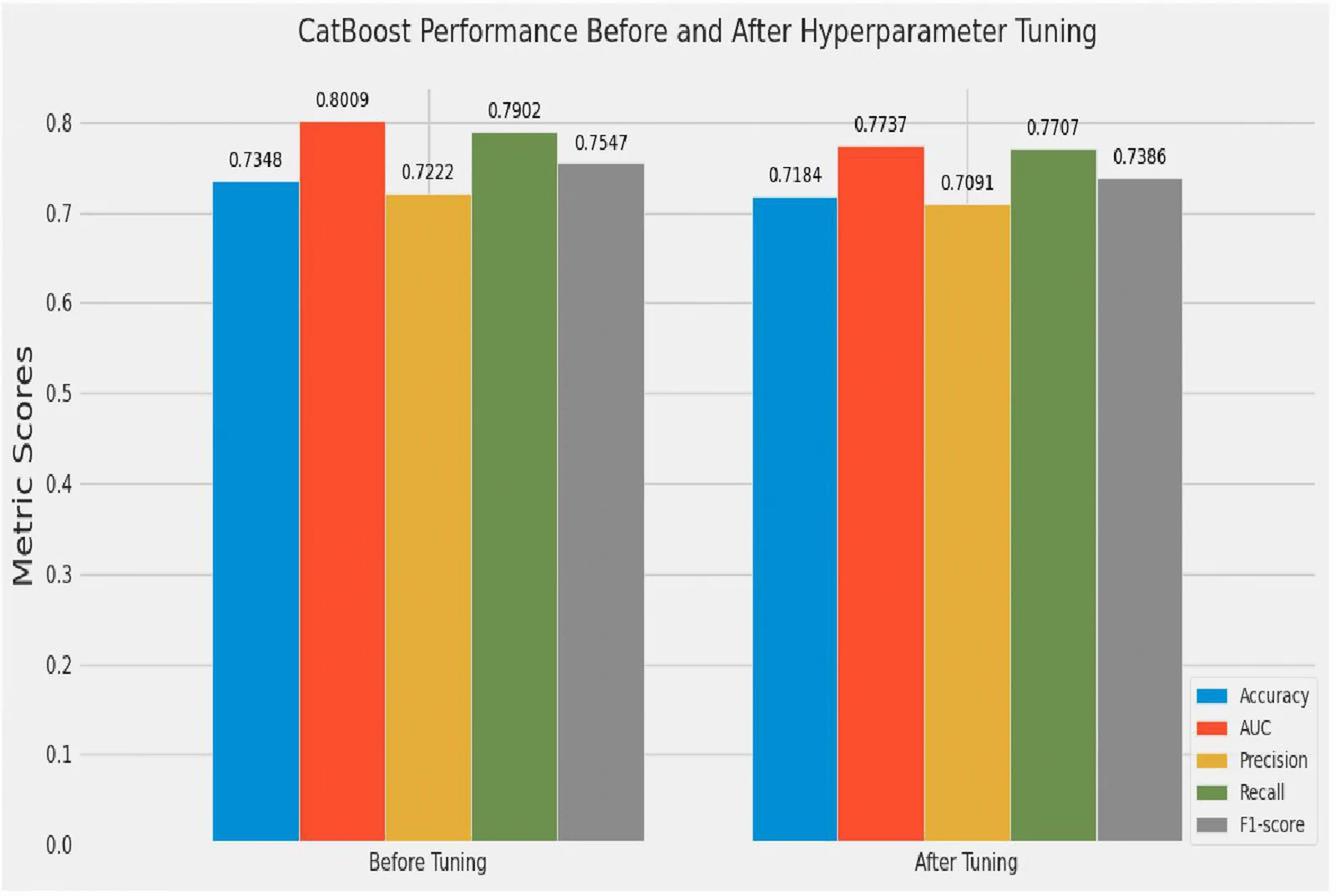
Comparison of before and after tuning with the Optuna framework

The Optuna framework’s hyperparameter tuning did not increase the performance of the balanced training dataset. Tuning often restricts depth or splits to avoid overfitting, but in ensemble models, this can reduce diversity and weaken predictive [31]. The Optuna hyperparameter search spaces that we used for CatBoost are presented in Table 6. The AUC was decreased from 80.09% to 77.37%, and accuracy decreased from 73.48% to 71.84%, as shown in Fig. 7. Therefore, the default (before tuning) balanced training dataset was used to test and predict the final model of intention to use contraceptives.

**Table 6:** Bayesian optimization, Optuna framework, hyperparameter tuning search space, and actual value for cat boosting

Parameters	Default search space	Actual search value
Learning rate	0.001–0.3	0.138
Maximum depth	3–15	12
L2 regularization	0.001–10	0.018
Bagging temperature	0.0–1	0.114
Border count	32–255	147
Number of trees	100–2000	125

### Leave-one-country-out cross-validation results

The leave-one-country-out analysis demonstrated moderate discriminative ability and acceptable transportability of the model across diverse national contexts. Overall performance metrics are summarized in Table 7. The mean weighted AUC was 0.74 ± 0.03, indicating consistent discrimination across countries. Accuracy averaged 0.67 ± 0.06, showing that approximately two-thirds of predictions were correct. Precision was more variable 0.67 ± 0.18, reflecting heterogeneity in false-positive rates across settings. Recall remained strong and relatively stable 0.74 ± 0.09, suggesting reliable identification of true positives. The F1 score, balancing precision and recall, averaged 0.68 ± 0.12, consistent with moderate overall performance. Taken together, these findings support the robustness of the model while highlighting variability in precision that warrants attention in country-specific applications.

**Table 7:** Overall leave-one-country-out cross-validation performance across 11 East African countries

Metric	Mean	Standard deviation
AUC	0.74	0.03
Accuracy	0.67	0.06
Precision	0.67	0.18
Recall	0.74	0.09
F1 Score	0.68	0.12

### Model calibration result

**Fig. 8:**
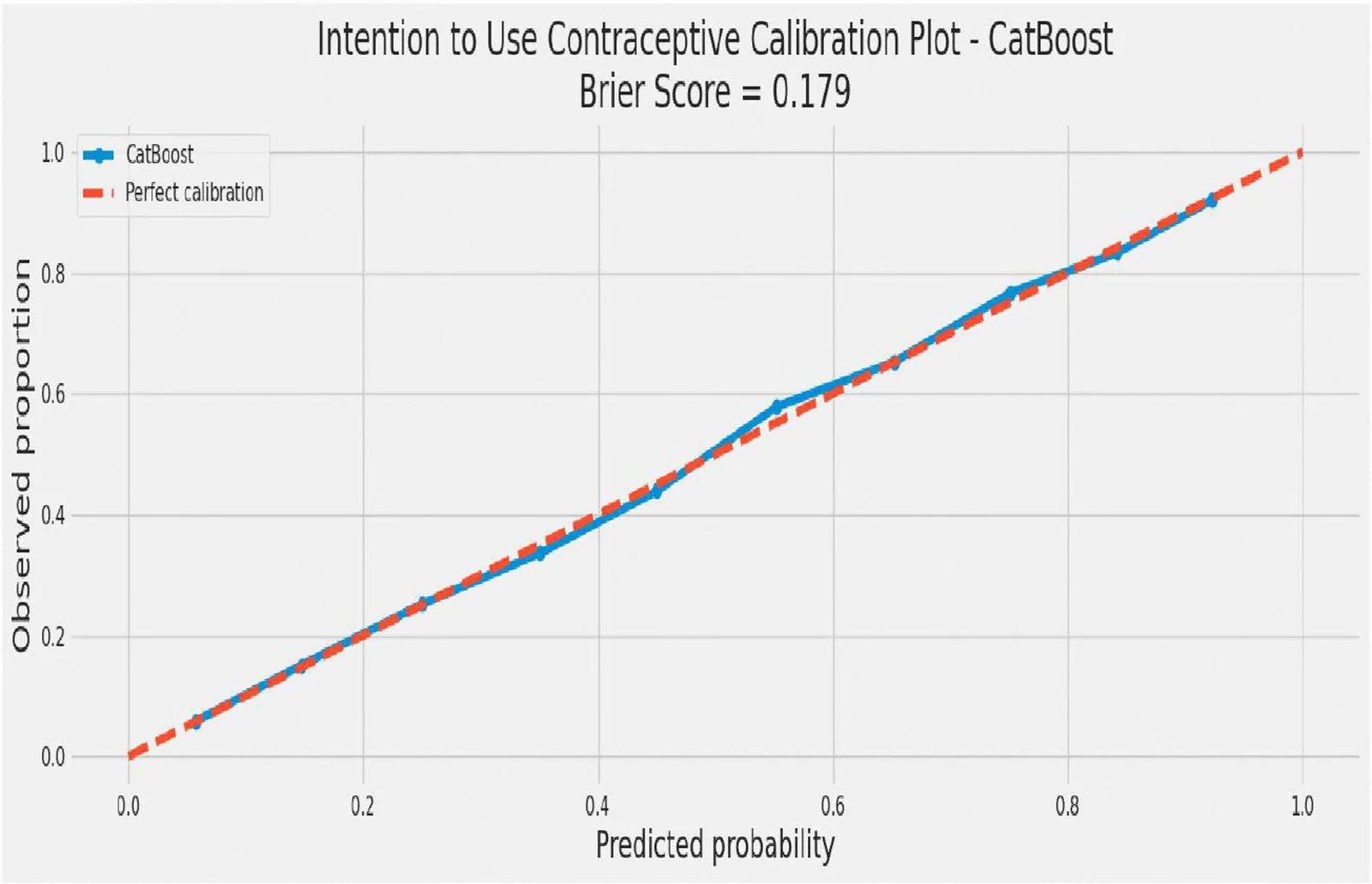
Calibration performance metrics of intention to use contraceptives using a cat boost model

In addition to discrimination metrics, we evaluated calibration to ensure the reliability of predicted probabilities for decision-support applications. As shown in Fig. 8, the CatBoost model achieved a Brier score of 0.179, and the calibration curve closely followed the 45° line of perfect calibration. This indicates that predicted probabilities were well aligned with observed outcomes, confirming that the model is not only discriminative (AUC = 0.8009) but also well calibrated. Together, these results strengthen confidence in the model’s suitability for classification tasks related to contraceptive intention.

### Prediction of intention to use a contraceptive

**Fig. 9:**
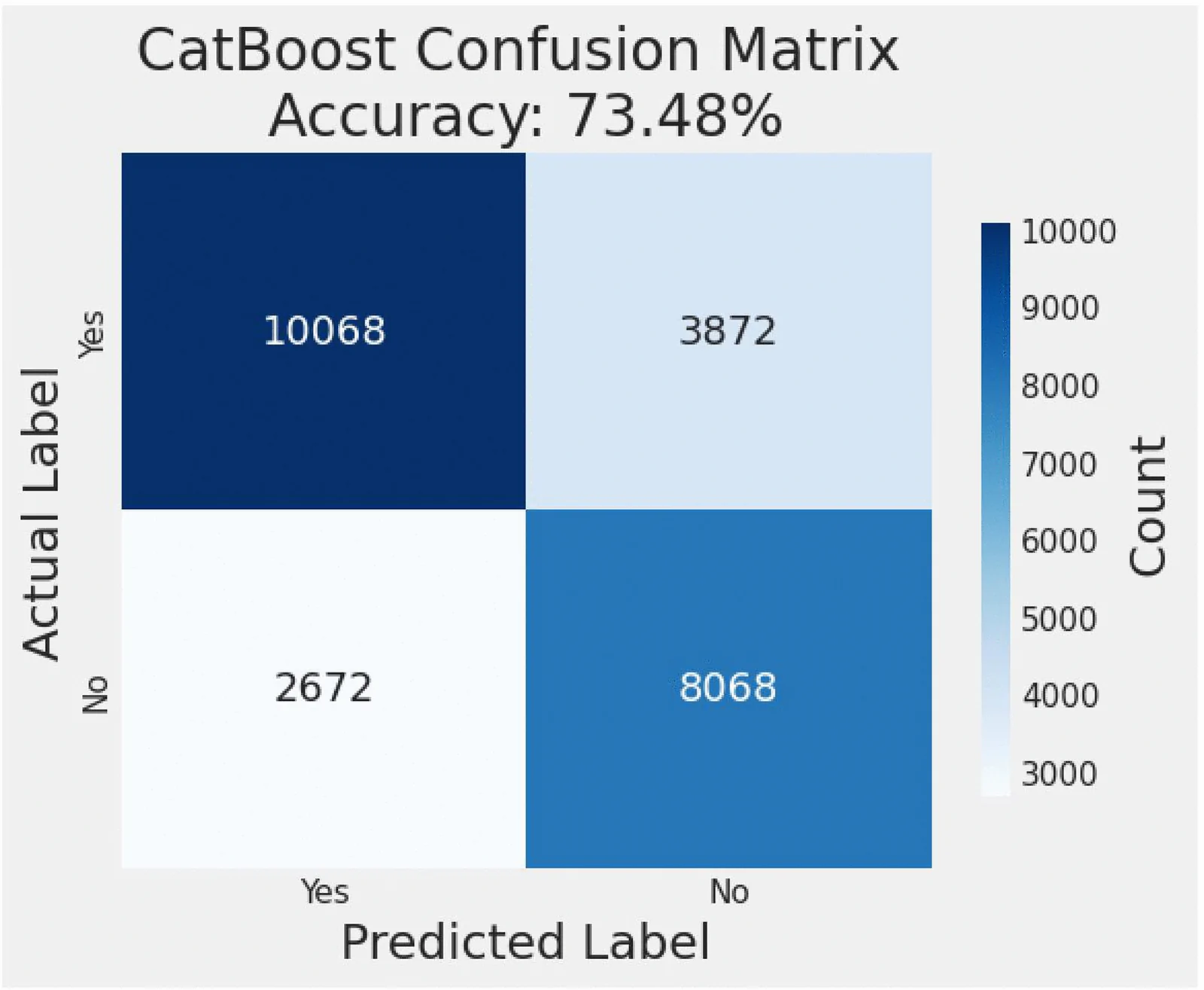
Prediction of intention to use contraceptives using a confusion matrix

The confusion matrix was used to evaluate the classification performance of the CatBoost model for predicting contraceptive intention. As shown in Fig. 9, the model correctly identified 10,068 women who reported intention to use contraceptives (true positives) and 8,068 women who did not intend to use contraceptives (true negatives). It misclassified 3,872 women who intended to use contraceptives as non-intenders (false negatives) and 2,672 women who did not intend to use contraceptives as intenders (false positives). This distribution illustrates the model’s ability to distinguish between intention and non-intention while also revealing areas of misclassification. From a policy perspective, false negatives represent missed opportunities to reach women receptive to contraceptive services, while false positives may lead to inefficient targeting. Together, the calibration and error analyses demonstrate that the model is both discriminative and well-calibrated, providing actionable predictions for guiding family-planning interventions and resource allocation.

### Global feature importance

**Fig. 10:**
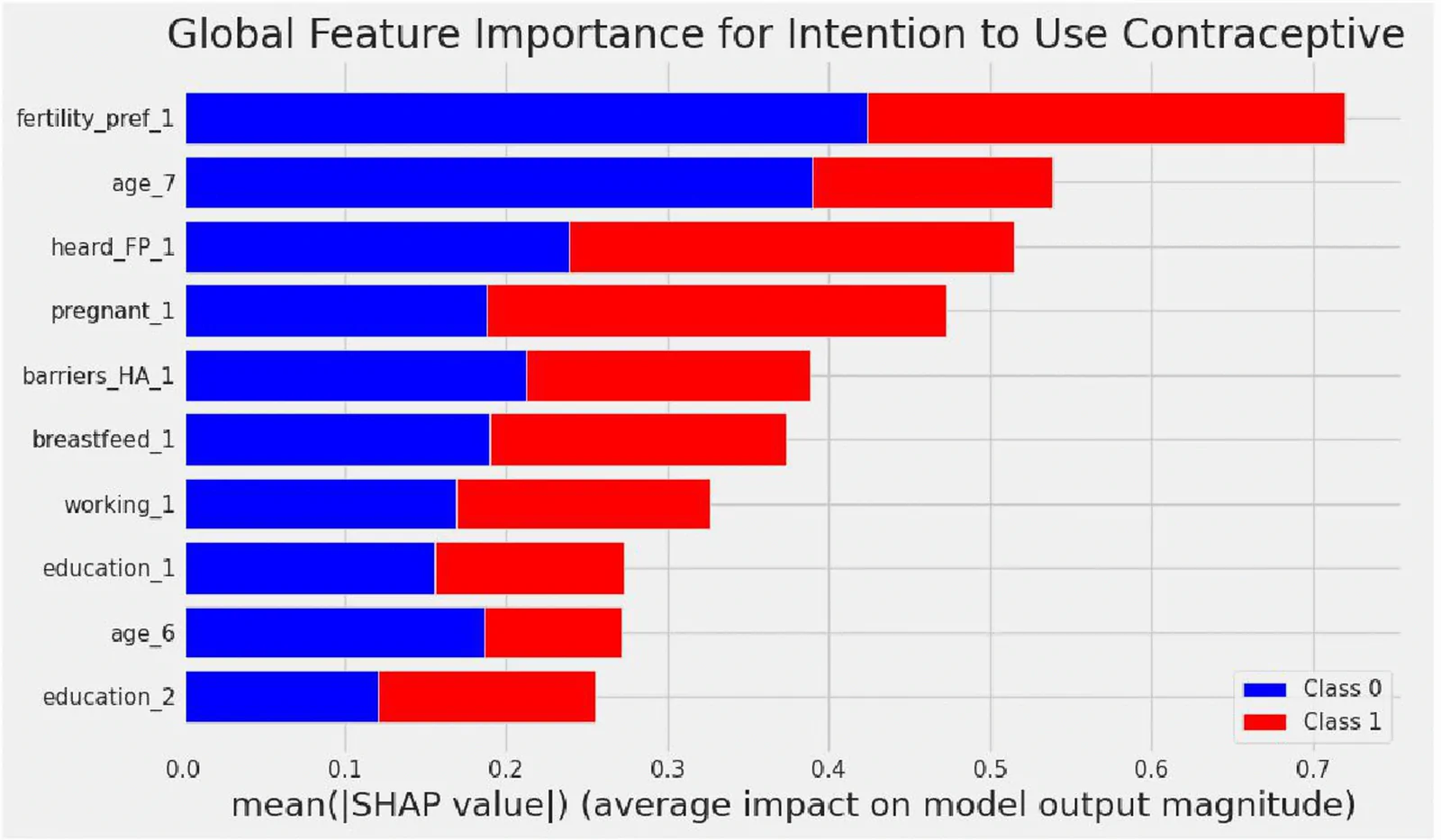
Global SHAP feature importance plot ranked by mean absolute value

The CatBoost machine-learning algorithm was applied to identify the most influential features contributing to predictions of contraceptive intention. SHAP values were calculated to quantify each feature’s contribution, and variables were ranked in descending order based on their mean absolute SHAP values. As illustrated in Fig. 10, the ten strongest predictors were fertility preferences, age 45–49 years, heard about family planning, current pregnancy status, barriers to healthcare access, current breastfeeding status, currently employed, attainment of primary education, age 40–45 years, and attainment of secondary education. These variables consistently demonstrated substantial predictive contributions to the model’s classification performance, underscoring the roles of reproductive experience, information exposure, socioeconomic position, and educational attainment in shaping contraceptive intention within the study population.

Each bar represents the average magnitude of a feature’s contribution to the predicted probability of contraceptive intention. Top predictors included fertility_pref_1 = had fertility preference, pregnant_1 = being currently pregnant, heard_FP_1 = heard about family planning, breastfeed_1 = being currently breastfeeding, age_7 = women aged 45–49, age_6 = women aged 40–44, working_1 = being employed, education_1 = being primary education level, education_2 = being secondary education level, barriers_HA_1 = experiencing barriers to healthcare access.

### Model interpretation

**Fig. 11:**
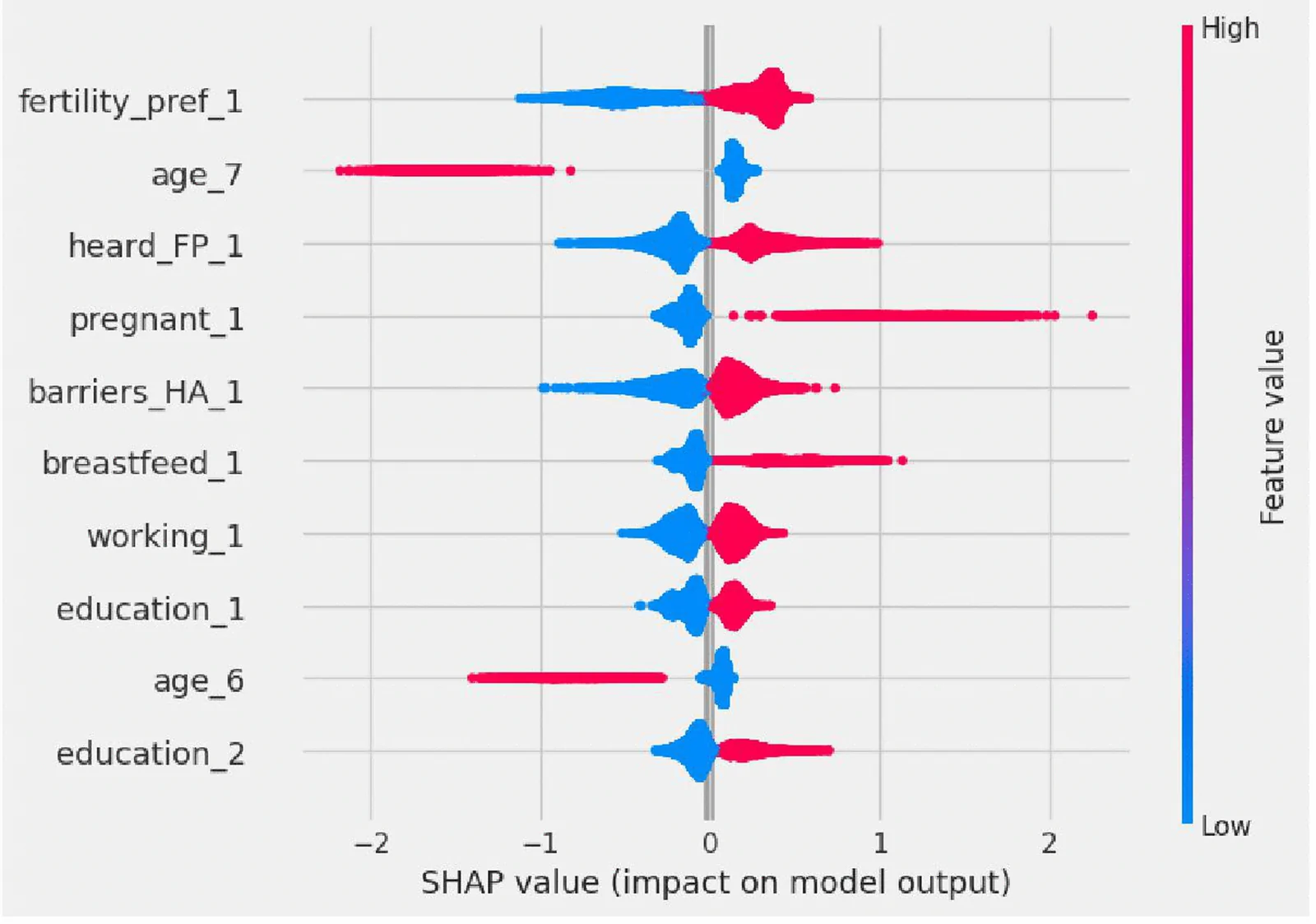
Beeswarm plot ranked by mean absolute SHAP value

The SHAP beeswarm plot provides a visual summary of how each feature contributed to the model’s predictions of contraceptive intention across the sample. Each point represents an individual, with color indicating the feature value (blue for low, red for high). The position on the X-axis reflects the direction and strength of the contribution: positive SHAP values (right) indicate higher predictive probability of intention, while negative SHAP values (left) indicate lower predictive probability. As shown in Fig. 11, several associated factors consistently showed positive contributions to contraceptive intention, including having fertility preferences, being currently pregnant, experiencing barriers to healthcare access, hearing about family planning, being currently breastfeeding, being employed, and attaining primary or secondary education. These features indicated that women with greater access to information, services, and education were more likely to be predicted by the model as intending to use contraceptive methods. Conversely, older age groups (40–44 and 45–49 years) were associated with negative SHAP values, reflecting lower predicted probabilities of intention.

Each dot represents an individual woman, with color indicating predictor value and horizontal position reflecting SHAP contribution to the predicted probability of contraceptive intention. Positive values indicate higher predicted probability; negative values indicate lower predicted probability. Top predictors included: fertility_pref_1 = had fertility preference, pregnant_1 = being currently pregnant, heard_FP_1 = heard about family planning, breastfeed_1 = being currently breastfeeding, age_7 = women aged 45–49, age_6 = women aged 40–44, working_1 = being employed status, education_1 = being primary education level, education_2 = being secondary education level, barriers_HA_1 = experiencing barriers to healthcare access.

### Local feature importance

**Fig. 12:**
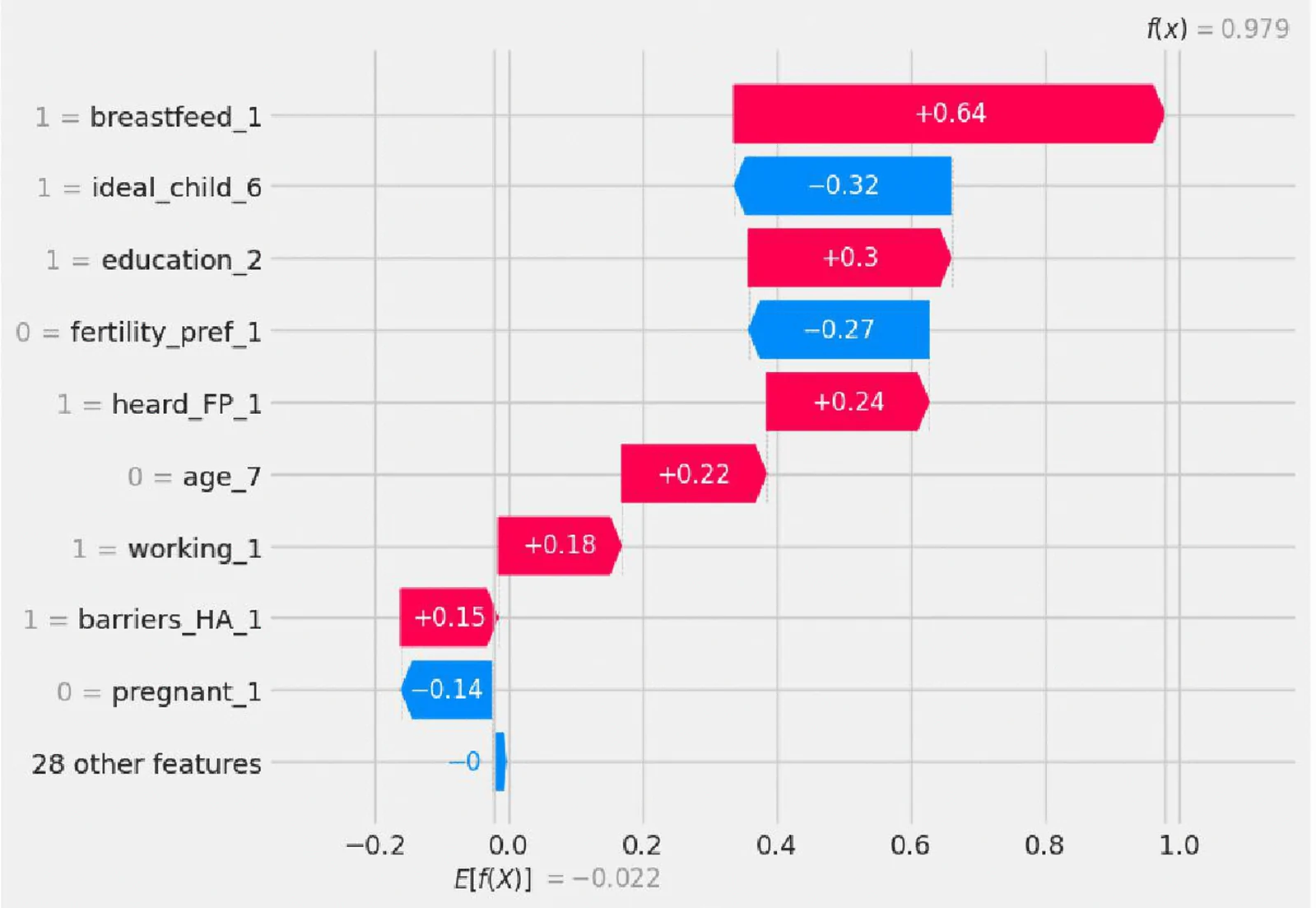
Waterfall plot for intention to use contraceptives

The local SHAP waterfall plot begins at the baseline prediction (E[f(x)] = -0.022) and shows how individual features contributed to the model’s output for this case. In this observation, as shown Fig. 12, features such as currently breastfeeding, having secondary education, hearing about family planning, being younger than 45–49 years, being employed, and experiencing barriers to healthcare access contributed positively to the predicted probability of contraceptive intention. Conversely, factors such as not being fertility-preferred, not currently pregnant, and reporting an ideal of six or more children contributed negatively. The final model output for this case was f(x) = 0.979, indicating a high predicted probability of intention to use contraceptives. We emphasize that SHAP values reflect contributions to prediction within the trained model and that local explanations are case-specific examples chosen to complement global feature importance. Together with global feature importance, these local explanations provide complementary insight into how the model differentiates intention from non-intention.

ach bar shows the contribution of a feature to the predicted probability of contraceptive intention relative to the baseline prediction. Positive values increase the predicted probability, while negative values decrease it. Variables are coded as: 1=breastfeed_1 = currently breastfeeding, 0=fertility_pref_1 = not fertility-preferred, 0=age_7 = not being aged 45–49 years, 1=education_2 = having secondary education status, 0=pregnant_1 = not currently pregnant, 1=ideal_6 = ideal have six or more children, 1=working_1 = being employed, 1=barriers_1 = experiencing barriers to health care access, and 1=heard_FP_1 = hearing about family planning.

## Discussion

The purpose of this study was to employ a machine-learning framework to predict the intention to use contraception and identify its key predictive features among women of reproductive age in East Africa. Using a robust pipeline comprising data integration, one-hot encoding, an 80/20 data split, and 10-fold cross-validation across multiple algorithms, the boosted tree models, particularly the CatBoost algorithm, demonstrated the highest predictive performance, yielding an AUC of 80.09% and an accuracy of 73.48%. In this pooled sample, the overall magnitude of intention to use contraception was 51.21%. This descriptive baseline is higher than the 37.66% reported in a broader SSA [13], as well as individual country estimates previously reported in Ghana (31.70%) [9], Mozambique (44.70%) [10], Pakistan (42.00%) [14], and Ethiopia (44.11%) [11]. These variations likely reflect differences in the pooled sample compositions, sample sizes, and study periods inherent to the specific DHS waves compiled across these studies [13]. Subgroup analyses stratified by DHS survey years (2015–2020 and 2021–2024) yielded I² values of 99.5% and 99.9%, respectively. These persistently high heterogeneity estimates indicate that survey year did not materially reduce variability, suggesting that cross-country contextual differences rather than temporal factors are the primary drivers of heterogeneity in contraceptive intention. Using SHAP values to interpret the final model, the top features for predicting contraceptive intention were identified as education level, employment status, age, breastfeeding status, fertility preference, current pregnancy status, barriers to healthcare access, and hearing about family planning.

Consistent with the predictive weight observed in the model, women who attained primary and secondary education levels demonstrated a higher likelihood of intending to use contraceptives compared to those with no formal education. This finding aligns with existing literature across SSA [13], East Africa [32], low and middle-income countries [33], Gambia [34], Benin [4], and Ethiopia [35]. Within a predictive framework, formal education often serves as a robust proxy for access to information; prior research suggests that educated women frequently report greater exposure to family planning resources through school curricula, printed materials, mass media, and digital platforms [4, 36]. Furthermore, literature indicates that formal education correlates strongly with increased literacy regarding the health and economic strains of unplanned pregnancies, alongside a clearer understanding of the planning benefits of contraception [34]. This educational stratification may also reflect underlying dynamics of healthcare autonomy; while uneducated women in similar datasets often rely heavily on spousal decision-making regarding reproductive health, higher educational attainment is frequently associated with greater individual autonomy to determine future contraceptive use [35].

Regarding economic features, the model identified employment status as a key predictor, with employed women demonstrating a higher likelihood of intending to use contraceptives compared to those who were unemployed. This finding is consistent with studies conducted in SSA [37], Ghana [38], and Benin [4]. Within a predictive framework, employment status often functions as a proxy for socioeconomic positioning; existing literature suggests that formal or informal occupations frequently co-occur with heightened financial independence and greater exposure to community health networks, which may align with more proactive reproductive tracking [38]. Furthermore, prior research associates independent income streams with a reduced financial barrier to accessing healthcare services, thereby stratifying employed individuals into a cohort with higher measurable intent [4]. Conversely, the model’s capacity to utilize employment as a complex stratifying feature is mirrored by divergent trends in the literature. For instance, a study in the Gambia [34] observed an inverse association, where employed women exhibited a lower intention to use contraception in the future compared to their counterparts. Literature accounts for this variance by noting that in certain contexts, working cohorts report distinct confounding challenges such as demanding schedules, perceived lower frequency of intercourse, or heightened concerns regarding method-related adverse effects, which shift their predictive weight within a model [34].

Regarding demographic features, the model identified women’s age as an important predictive feature of contraceptive intention. Specifically, women in the older age cohorts, those aged 40–44 and 45–49 years, demonstrated a lower likelihood of intending to use contraceptives compared to the adolescent baseline group of 15–19 years. This finding is consistent with prior studies conducted in Cameron [39], Gambia [34], Uganda [40], Malawi [41], and Ethiopia [11]. This is connected to the fact that older women might not require contraceptive methods as frequently [16]. Existing literature suggests that older cohorts within cross-sectional datasets frequently encompass individuals who have completed their desired family size, entered menopausal or subfertility states, or already adopted long-term contraceptive methods before the survey administration, thereby altering their baseline intention status [4]. Conversely, localized research in Addis Ababa [42] and Debre Markos [43] noted an alternate trend, where an age greater than 30 years at first contraceptive use was associated with an increased probability of contraceptive intention. Literature attributes this variance to a threshold effect, where older women approaching the end of their reproductive years, having achieved their target parity, exhibit a highly concentrated intent to limit further childbearing [17].

According to this study, hearing about family planning was identified as an important predictive feature associated with the intention to use contraceptives. Women who had heard about family planning demonstrated a higher probability of intending to use contraceptives compared to their counterparts. This finding is supported by studies conducted in Jordan [44], SSA [39], and Benin [4, 45]. Within an associative framework, hearing about family planning via media channels serves as a structural indicator of informational access. Existing literature suggests that populations exposed to reproductive health messaging frequently exhibit a higher baseline understanding of contraceptive options, which correlates strongly with receptive attitudes toward future method adoption [46]. Furthermore, prior research notes that consistent communication regarding reproductive health intersects with fewer informational and socio-cultural barriers to healthcare service utilization [4].

According to this study, breastfeeding was identified as an important predictive feature associated with the intention to use contraceptives. Women who are currently breastfeeding are more likely to intend to use contraceptives compared to women who are not currently breastfeeding. Within a predictive framework, current breastfeeding status often serves as a structural indicator of an active postpartum life stage where contact with maternal healthcare systems is heightened. Existing literature suggests that breastfeeding cohorts frequently intersect with populations prioritizing birth spacing or those who have had recent exposure to postpartum family planning counseling [47].

The model also identified current pregnancy status as a prominent predictive feature of a woman’s intention to use contraceptives, with pregnant women demonstrating a higher probability of contraceptive intent compared to non-pregnant women. This finding is consistent with research done in Adigrat [48] and Aksum [16]. Within an associative framework, a current pregnancy often serves as a powerful indicator of immediate, ongoing navigation through maternal healthcare systems. Existing literature suggests that pregnant cohorts frequently encompass individuals reflecting on prior delivery experiences or those who have achieved their target family size, thereby concentrating their expressed interest in subsequent birth spacing or limiting further parity [49]. Furthermore, prior research indicates that women navigating a current pregnancy represent a sub-population with highly formalized contact with antenatal and maternal health services [50]. This heightened engagement throughout the continuum of care structurally embeds these individuals within networks where postpartum contraceptive counseling is heavily integrated.

This study revealed that fertility preference was identified as an important predictive feature associated with the intention to use contraceptives. Women who had fertility preferences were more likely to have the intention to use contraceptives compared to their counterparts. This finding is supported by a study conducted in Uganda [51]. Within a predictive framework, stated fertility preferences operate as a powerful marker of structured reproductive planning, where a desire to space births over a longer interval frequently aligns with higher baseline tracking of family planning options [51]. Conversely, a broader study in Sub-Saharan Africa [13] identified a more nuanced relationship, capturing instances where fertility desires did not perfectly align with contraceptive intentions. Existing literature accounts for this variance by noting unmeasured interpersonal and contextual confounders within pooled cross-sectional data. For instance, a male partner’s distinct childbearing preferences can intersect with and heavily alter a woman’s individual reproductive decisions [52]. Furthermore, empirical research demonstrates that fertility preferences are fluid rather than static; evolving partner relationships, shifting economic needs, and unmeasured lifecycle events continuously reshape a woman’s motivation to space or restrict subsequent births [51].

According to this study, barriers to healthcare access were identified as an important predictive feature associated with the intention to use contraceptives. Women who experienced barriers to healthcare access were more likely to report an intention to use contraception compared to those who did not face such barriers. Existing literature suggests that when physical or financial services are difficult to obtain, individuals frequently signal underlying demand through stated future intentions, anticipating adoption once structural barriers are reduced [53]. Furthermore, research notes that socio-cultural challenges or structural limitations often stratify populations into cohorts with concentrated future planning profiles [54], as communities confronting deep structural constraints frequently express robust latent demand despite delayed actual uptake [55].

### Strengths and limitations of the study

This study has several notable strengths. The use of a large, multi-country dataset provides broad coverage across diverse demographic and contextual settings, which enhances the representativeness of the findings. In addition, the attempt at model interpretability through SHAP analysis is a valuable contribution, as it allows readers to understand the relative contribution of predictors to model performance. However, this study has some limitations. Substantial cross-country heterogeneity in contraceptive intention persisted even after subgroup analyses. The surveys were conducted across different years; temporal confounding cannot be fully excluded, even though stratified analyses by survey period (2015–2020 vs. 2021–2024) showed that heterogeneity remained high. The absence of a regression coefficient made it difficult to use magnitude to interpret the strength of association between the predictor variables and intention to use contraceptives. Furthermore, because the data and surveys are cross-sectional, the study’s findings do not show a cause-and-effect relationship between the outcome variable and the independent variables.

## Conclusion

In this pooled analysis of DHS data from 11 East African countries, boosted tree models, particularly CatBoost, demonstrated moderate discrimination in predicting women’s intention to use contraception. Key predictors included education, employment, age, fertility preference, breastfeeding, pregnancy status, barriers to healthcare access, and exposure to family planning information. This study provides a transparent, reproducible framework for identifying risk patterns in contraceptive intention across diverse settings and serves as a foundation for future applied work.

## Supplementary Information

Supplementary Material 1. S1 Table TRIPOD-AI reporting guideline checklist.

## Data Availability

The data for this study were obtained from the EDHS Program. These datasets are publicly available and can be requested from the DHS website at https://dhsprogram.com/data/dataset_admin/index.cfm.

## Abbreviations

AUC: Area under the Curve
DHS: Demographic Health Survey
SHAP: Shapely Additive Explanation
ROC: Receiver Operating Characteristic

## References

[1] Jain R, Muralidhar S. Contraceptive methods: needs, options and utilization. J Obstet Gynecol India. 2011;61(6):626–34. 10.1007/s13224-011-0107-7 PMC3307935 23204678

[2] Gebeyehu NA, et al. The Intention on Modern Contraceptive Use and Associated Factors among Postpartum Women in Public Health Institutions of Sodo Town, Southern Ethiopia 2019: An Institutional-Based Cross‐Sectional Study. Biomed Res Int. 2020;2020(1):9815465. 10.1155/2020/9815465 PMC7568156 33102602

[3] Magalona S, et al. Contraceptive intentions and use throughout the extended postpartum period: a panel study in Ethiopia. Stud Fam Plann. 2023;54(4):543–62. 10.1111/sifp.12252 37845821

[4] Kuug AK, et al. Assessment of intention to use modern contraceptives among women of reproductive age in Benin: evidence from a national population-based survey. Contracept Reproductive Med. 2024;9(1):43. 10.1186/s40834-024-00294-8 PMC11363446 39215322

[5] Solanke BL, et al. Do health service contacts with community health workers influence the intention to use modern contraceptives among non-users in rural communities? Findings from a cross-sectional study in Nigeria. BMC Health Serv Res. 2023;23(1):24. 10.1186/s12913-023-09032-3 PMC9832820 36627614

[6] Up AI. Investing in Contraception and Maternal and Newborn Health, 2017. Guttmacher Institute; 2017.

[7] Organization WH. WHO recommendations on self-care interventions: self-administration of injectable contraception. World Health Organization; 2020.

[8] Boadu I. Coverage and determinants of modern contraceptive use in sub-Saharan Africa: further analysis of demographic and health surveys. Reproductive Health. 2022;19(1):18. 10.1186/s12978-022-01332-x PMC8781110 35062968

[9] Bawah AA, et al. Contraceptive use intentions and unmet need for family planning among reproductive-aged women in the Upper East Region of Ghana. Reproductive health. 2019;16(1):26. 10.1186/s12978-019-0693-x PMC6399919 30832695

[10] Tekelab T, Sufa A, Wirtu D. Factors affecting intention to use long-acting and permanent contraceptive methods among married women of reproductive age groups in Western Ethiopia: a community-based cross-sectional study. Fam Med Med Sci Res. 2015;4(158):2.

[11] Gilano G, Hailegebreal S. Assessment of intention to use contraceptive methods with spatial distributions and associated factors among women in Ethiopia: evidence from EDHS 2016. Archives Public Health. 2021;79(1):109. 10.1186/s13690-021-00631-2 PMC8215759 34154673

[12] Yeboah D, et al. Prevalence and factors associated with the intention to use contraception among women of reproductive age who are not already using a contraceptive method in Liberia: findings from a secondary analysis of the 2019–2020 Liberia Demographic Health Survey. BMJ Open. 2023;13(10):e072282. 10.1136/bmjopen-2023-072282 PMC10565247 37802625

[13] Negash WD, Eshetu HB, Asmamaw DB. Intention to use contraceptives and its correlates among reproductive age women in selected high fertility sub-Saharan Africa countries: a multilevel mixed effects analysis. BMC Public Health. 2023;23(1):257. 10.1186/s12889-023-15187-9 PMC9901088 36747157

[14] Agha S. Intentions to use contraceptives in Pakistan: implications for behavior change campaigns. BMC Public Health. 2010;10(1):450. 10.1186/1471-2458-10-450 PMC2920282 20673374

[15] Meskele M, Mekonnen W. Factors affecting women’s intention to use long-acting and permanent contraceptive methods in Wolaita Zone, Southern Ethiopia: A cross-sectional study. BMC Womens Health. 2014;14(1):109. 10.1186/1472-6874-14-109 PMC4237819 25216640

[16] Abraha TH, Belay HS, Welay GM. Intentions on contraception use and its associated factors among postpartum women in Aksum town, Tigray region, northern Ethiopia: a community-based cross-sectional study. Reproductive health. 2018;15(1):188. 10.1186/s12978-018-0632-2 PMC6234798 30413214

[17] Adem JB, et al. Explainable machine learning algorithms to identify predictors of intention to use family planning among women of reproductive age in Ethiopia: Evidence from the Performance Monitoring and Accountability (PMA) 2021 survey data set. BMJ Public Health. 2025;3(1). 10.1136/bmjph-2024-000962 PMC12185885 40557287

[18] Götmark F, Andersson M. Human fertility in relation to education, economy, religion, contraception, and family planning programs. BMC Public Health. 2020;20(1):265. 10.1186/s12889-020-8331-7 PMC7036237 32087705

[19] Liu F, Panagiotakos D. Real-world data: a brief review of the methods, applications, challenges and opportunities. BMC Med Res Methodol. 2022;22(1):287. 10.1186/s12874-022-01768-6 PMC9636688 36335315

[20] Birlie AA, et al. Classification of knowledge of fertility period among adolescent girls in East Africa from 2012 to 2022: Machine learning algorithm. PLOS Digit Health. 2026;5(2):e0001108. 10.1371/journal.pdig.0001108 PMC12928492 41729861

[21] Tadese ZB, et al. Exploring machine learning algorithms for predicting fertility preferences among reproductive age women in Nigeria. Front Digit Health. 2025;6:1495382. 10.3389/fdgth.2024.1495382 PMC11781225 39886062

[22] Irmalasari I, Dwiyanti L. Algorithm analysis of decision tree, gradient boosting decision tree, and random forest for classification (Case Study: West Java House of Representatives Election 2019). In the 2023 International Conference on Electrical Engineering and Informatics (ICEEI). IEEE; 2023.

[23] Dev VA, Eden MR. Gradient boosted decision trees for lithology classification, in Computer-aided chemical engineering. Elsevier; 2019. pp. 113–8.

[24] Azmi SS, Baliga S. An overview of boosting decision tree algorithms utilizing AdaBoost and XGBoost boosting strategies. Int Res J Eng Technol. 2020;7(5):6867–70.

[25] Pedregosa F, et al. Scikit-learn: Machine learning in Python. J Mach Learn Res. 2011;12:2825–30.

[26] Melaku MS, et al. Application of machine learning algorithms to model predictors of informed contraceptive choice among reproductive age women in six high fertility rate sub-Saharan Africa countries. BMC Public Health. 2025;25(1):1986. 10.1186/s12889-025-23242-w PMC12121055 40442626

[27] Bifarin OO. Interpretable machine learning with tree-based Shapley additive explanations: Application to metabolomics datasets for binary classification. PLoS ONE. 2023;18(5):e0284315. 10.1371/journal.pone.0284315 PMC10159207 37141218

[28] Nohara Y, et al. Explanation of machine learning models using Shapley additive explanation and application for real data in a hospital. Comput Methods Programs Biomed. 2022;214:106584. 10.1016/j.cmpb.2021.106584 34942412

[29] Asnake AA, et al. Application of machine learning algorithm for prediction of abortion among reproductive age women in Ethiopia. Sci Rep. 2025;15(1):17924. 10.1038/s41598-025-95342-x PMC12102202 40410396

[30] Collins GS, et al. TRIPOD + AI statement: updated guidance for reporting clinical prediction models that use regression or machine learning methods. BMJ. 2024;385. 10.1136/bmj-2023-078378 PMC11019967 38626948

[31] Fernández-Delgado M, et al. Do we need hundreds of classifiers to solve real-world classification problems? J Mach Learn Res. 2014;15(1):3133–81.

[32] Bakibinga P, et al. Pregnancy history and current use of contraception among women of reproductive age in Burundi, Kenya, Rwanda, Tanzania, and Uganda: analysis of demographic and health survey data. BMJ Open. 2016;6(3):e009991. 10.1136/bmjopen-2015-009991 PMC4800125 26966059

[33] Blumenberg C, et al. Socio-demographic and economic inequalities in modern contraception in 11 low-and middle-income countries: an analysis of the PMA2020 surveys. Reproductive health. 2020;17(1):82. 10.1186/s12978-020-00931-w PMC7268403 32487182

[34] Enyew EF, Jembere MM, Terefe B. Intention to Use Contraceptives and Determinants Among Married Women in Gambia: A Further Analysis of the National Survey. Sage Open. 2025;15(4):21582440251395796.

[35] Alemayehu M, et al. Family planning use and associated factors among the pastoralist community of the Afar region, eastern Ethiopia. BMC Womens Health. 2016;16(1):39. 10.1186/s12905-016-0321-7 PMC4950765 27430275

[36] Breuner CC, et al. Sexuality education for children and adolescents. Pediatrics. 2016;138(2). 10.1542/peds.2016-1348 27432844

[37] Budu E, et al. Intention to use contraceptives among married and cohabiting women in sub-Saharan Africa: a multilevel analysis of cross-sectional data. BMJ Open. 2022;12(11):e060073. 10.1136/bmjopen-2021-060073 PMC9693891 36424119

[38] Ahuja M, et al. Risk and protective factors for intention of contraception use among women in Ghana. Health Psychol Open. 2020;7(2):2055102920975975. 10.1177/2055102920975975 PMC7720324 33329897

[39] Rai RK. Future intention of contraceptive use among Comorian women, 2012. J Public Health. 2015;23(5):289–96.

[40] Lutalo T, et al. Unfulfilled need for contraception among women with unmet need but with the intention to use contraception in Rakai, Uganda: a longitudinal study. BMC Womens Health. 2018;18(1):60. 10.1186/s12905-018-0551-y PMC5921782 29699548

[41] Forty J, Rakgoasi SD, Keetile M. Patterns and determinants of modern contraceptive use and intention to use contraceptives among Malawian women of reproductive age (15–49 years). Contracept reproductive Med. 2021;6(1):21. 10.1186/s40834-021-00163-8 PMC8247247 34193289

[42] Nesro J, et al. Intention to use vasectomy and associated factors among married men in Addis Ababa, Ethiopia. BMC Public Health. 2020;20(1):1228. 10.1186/s12889-020-09316-x PMC7424675 32787807

[43] Abajobir AA. Intention to use long-acting and permanent family planning methods among married 15–49-year-old women in Debremarkos Town, Northwest Ethiopia. Fam Med Med Sci Res. 2014;3(145):2.

[44] Alomair N, et al. Factors influencing sexual and reproductive health of Muslim women: a systematic review. Reproductive health. 2020;17(1):33. 10.1186/s12978-020-0888-1 PMC7059374 32138744

[45] Solanke BL, et al. Maternal grand multiparity and intention to use modern contraceptives in Nigeria. BMC Public Health. 2018;18(1):1207. 10.1186/s12889-018-6130-1 PMC6206733 30373559

[46] Asmamaw DB, Eshetu HB, Negash WD. Individual and community-level factors associated with intention to use contraceptives among reproductive age women in sub-Saharan Africa. Int J public health. 2022;67:1604905. 10.3389/ijph.2022.1604905 PMC9278060 35845431

[47] Loewenberg Weisband Y, et al. Postpartum intentions on contraception use and method choice among breastfeeding women attending a university hospital in Ohio: a cross-sectional study. Reproductive Health. 2017;14(1):45. 10.1186/s12978-017-0307-4 PMC5360022 28320478

[48] Gebremariam A, Addissie A. Intention to use long-acting and permanent contraceptive methods and factors affecting it among married women in Adigrat town, Tigray, Northern Ethiopia. Reproductive health. 2014;11(1):24. 10.1186/1742-4755-11-24 PMC4007570 24628764

[49] Rusibamayila A, et al. Factors influencing pregnancy intentions and contraceptive use: an exploration of the ‘unmet need for family planning’ in Tanzania. Cult Health Sex. 2017;19(1):1–16. 10.1080/13691058.2016.1187768 27297661

[50] Mekonnen BD, Vasilevski V. Effect of pregnancy intention on completion of maternity continuum of care in Sub-Saharan Africa: systematic review and meta-analysis. 2024;24(1):802. 10.1186/s12884-024-06998-8 PMC11603981 39609727

[51] Sarnak DO, et al. A Panel Study of Fertility Preferences and Contraceptive Dynamics in the Presence of Competing Pregnancy Risks in Uganda. Demography. 2021;58(1):295–320. 10.1215/00703370-8937285 33834246

[52] Yeatman S, Sennott C. The sensitivity of measures of unwanted and unintended pregnancy using retrospective and prospective reporting: Evidence from Malawi. Matern Child Health J. 2015;19(7):1593–600. 10.1007/s10995-015-1669-2 PMC4476926 25636647

[53] Bawuah A, et al. Understanding contraception-use intentions among women of reproductive age not currently using contraceptives in sub-Saharan Africa: key insights from Demographic and Health Surveys. Int Health. 2026;18(1):48–60. 10.1093/inthealth/ihaf053 PMC12766447 40390684

[54] Schulte A, et al. Relationship between experiencing a challenge or delay accessing contraception and contraceptive self-efficacy: Data from a 2022 nationally representative online survey. Reproductive Health. 2025;22(1):54. 10.1186/s12978-025-02003-3 PMC12001391 40234968

[55] Hart J. Reshaping contraceptive access efforts by centering equity, justice, and autonomy. 2022, American Public Health Association. pp. S468–S468. 10.2105/AJPH.2022.306926 PMC10490310 35767783

