# Supplementary material for: Predicting women’s intention to use contraceptives in East Africa: a machine learning analysis of predictors
Source: BMC Womens Health. 2026 Jul 20;26:544. doi: 10.1186/s12905-026-04705-7 (PMC13685393; doi:10.1186/s12905-026-04705-7)
Supplement: Supplementary file 1 [file 12905_2026_4705_MOESM1_ESM.docx]

**S1** **Table** TRIPOD-AI reporting guideline checklist

| **Section** | **Item** | **Requirement** | **Study Response** |
| --- | --- | --- | --- |
| **Title & Abstract** | Title | Identify the study as a prediction model, specify the AI/ML method | The title states “Predicting women’s intention to use contraceptives in East Africa: A machine learning analysis of predictors” and specifies CatBoost as the best model. |
|  | Abstract | Summarize objectives, data, participants, predictors, outcome, model type, validation, and performance. | Abstract includes DHS dataset (2015-2024, 11 countries, N=123,290), predictors, outcome, ML algorithms, LOCO‑CV, and CatBoost performance (AUC=80.09%). |
| **Introduction** | Background | Explain the medical context and rationale | The introduction explains the reproductive health burden, contraceptive gaps, and rationale for ML prediction. |
|  | Objectives | State study aims | Aim: to predict contraceptive intention among reproductive‑age women in East Africa using ML. |
| **Methods** | Data source | Describe dataset(s), setting, timeframe | DHS datasets from 11 East African countries, 2015-2024. |
|  | Participants | Eligibility criteria | Reproductive‑age women (15-49), N=123,290, after exclusions and weighting. |
|  | Outcome | Define outcome | DHS contraceptive intention item; “unsure” recoded as “no intention.” |
|  | Predictors | Define predictors and missing data handling. | Sociodemographic, fertility, FP exposure, breastfeeding, barriers, education, employment; missing data imputed with k‑NN. |
|  | Sample size | Justify the sample size | Large DHS dataset (N=123,290), nationally representative. |
|  | Model development | Algorithms, hyperparameters, feature selection | Seven ML algorithms tested; CatBoost tuned with Optuna; default parameters retained. |
|  | Validation | Internal/external validation | Train/test split; leave‑one‑country‑out cross‑validation. |
|  | Performance measures | Metrics reported | Accuracy, precision, recall, F1, ROC‑AUC; calibration (Brier score). |
|  | Explainability | Model interpretation | SHAP feature importance, beeswarm, and waterfall plots are used for interpretation. |
| **Results** | Participants | Flow diagram, baseline characteristics | Tables of sociodemographic and reproductive characteristics are reported. |
|  |  | Descriptive data | Prevalence 51.23% overall; country range 25.89–72.13% |
|  | Model development | Final model specification | CatBoost was identified as the best model; predictors were retained. |
|  | Performance | Report metrics with variability | CatBoost AUC=80.09%, accuracy=73.48%, precision=72.22%, recall=79.02%, F1=75.47; LOCO‑CV mean AUC=0.74 (SD=0.03). |
|  | Validation results | Internal and external validation | 10-fold CV for internal and LOCO‑CV for external validation |
|  | Key predictors | Top features | Fertility preference, age, FP exposure, pregnancy, breastfeeding, barriers, employment, education |
| **Discussion** | Limitations | Bias, generalizability | Limitations noted: high heterogeneity, lack of regression coefficient; pooled SHAP values reflect regional associations. |
|  | Interpretation | Compare with prior studies | Findings consistent with DHS literature; ML adds predictive efficiency. |
|  | Implications | Practical use, policy relevance | Identifies predictors of contraceptive intention; informs targeted interventions; causal claims tempered. |
| **Other Information** | Supplementary materials | Code, artefacts, repository links | Scripts and artefacts were deposited in a public repository (GitHub). |
|  | Funding | Sources of support | No fund |
|  | Conflicts of interest | Declare any | The authors declare that they have no. |
|  | Checklist submission | Completed checklist submitted | Checklist prepared and submitted as supplementary material. |
